# The Impact of a Polyphenol-Rich Extract from the Berries of *Aronia melanocarpa* L. on Collagen Metabolism in the Liver: A Study in an In Vivo Model of Human Environmental Exposure to Cadmium

**DOI:** 10.3390/nu12092766

**Published:** 2020-09-10

**Authors:** Magdalena Kozłowska, Małgorzata M. Brzóska, Joanna Rogalska, Anna Galicka

**Affiliations:** 1 Department of Toxicology, Medical University of Bialystok, Adama Mickiewicza 2C street, 15-222 Bialystok, Poland; joanna.rogalska@umb.edu.pl; 2 Department of Medical Chemistry, Medical University of Bialystok, Adama Mickiewicza 2A street, 15-222 Bialystok, Poland; angajko@umb.edu.pl

**Keywords:** *Aronia melanocarpa* L., cadmium, chokeberry, collagen, liver, matrix metalloproteinases, polyphenols, protection, tissue metalloproteinase inhibitors

## Abstract

This study examined whether a polyphenol-rich extract from the berries of *Aronia melanocarpa* L. (AE; chokeberries) may protect from the impact of cadmium (Cd) on the metabolism of collagen in the liver. The study was conducted in an experimental model (rats that were fed a diet containing 1 or 5 mg Cd/kg for 3–24 months) of human exposure to this xenobiotic during a lifetime. The concentration of total collagen and the expression of collagen types I and III at the mRNA and protein levels, as well as the concentrations of matrix metalloproteinases (MMP-1 and MMP-2) and their tissue inhibitors (TIMP-1 and TIMP-2), were assayed. The administration of Cd and/or AE had only a slight and temporary impact on the concentration of total collagen in the liver. The supplementation with AE significantly prevented Cd-mediated changes in the expression of collagen types I and III at the mRNA and protein levels and their ratio (collagen III/collagen I), as well as a rise in the concentrations of MMPs and TIMPs in this organ. The results allow the conclusion that the intake of chokeberry products in the case of Cd intoxication may be effective in prevention from this xenobiotic-induced disturbance in collagen homeostasis in the liver.

## 1. Introduction

Polyphenols are a wide group of natural compounds present in numerous food and medicinal plants [1,2]. These compounds are characterized by proven beneficial actions in organisms, such as antioxidative, anti-inflammatory, antiatherogenic, antidiabetic, and chelating properties [1,2,3,4,5,6]. Owing to this fact, the consumption of polyphenol-rich products is widely recommended, not only by nutritionists. For some time, there has been a growing interest in the opportunity of the use of plant-based items rich in these compounds in prophylaxis and to support the treatment of many diseases, including civilization diseases [2,5,6]. Currently, special attention has been paid to potential preventive strategies against the health outcomes of intoxication with various xenobiotics, including cadmium (Cd), to which the general population is exposed during the lifetime; treatment of these has also been focused on polyphenols and products abundant in these compounds [1,2,7,8,9]. Based on the available experimental data, it is well known that polyphenols attenuate the toxic effects of heavy metals, including Cd (for review, see [1,2,3,9]); however, it is very important to find the most effective polyphenolic compound or polyphenol-rich product that would be a promising candidate for its possible further use in humans.

Cd is one of the most dangerous contaminants of food and the environment in economically developed countries, and it is predicted that exposure to this heavy metal will increase [7,8,9]. Furthermore, more numerous data show that even low-level long-term intoxication with this toxic metal creates a threat to the health of the general population, contributing to the development of numerous unfavorable health effects [8,9,10,11]. Therefore, searching for an effective method of protection from Cd toxicity is a main focus of researchers.

Among food products, polyphenol-rich items, such as berries of *Aronia melanocarpa* L. (chokeberries; [Michx.] Elliott, *Rosaceae*), are of special interest in relation to protection from the detrimental effects of exposure to xenobiotics acting as pro-oxidants, including Cd [1,2,9,12,13,14,15,16,17]. Owing to the high content of hydroxyl groups (-OH groups), polyphenolic compounds can form complexes with ions of divalent elements, including ions of Cd (Cd^2+^), and can detoxify reactive oxygen species (ROS) and free radicals (FR) [1,2,3,12,13,14]. We have already reported that, in an experimental model of low and moderate human exposure to this xenobiotic during a lifetime (female rats maintained on a diet containing 1 and 5 mg Cd/kg, respectively, for 3–24 months), an extract from *A. melanocarpa* berries (AE) prevented the unfavorable influence of this element on the skeletal system, liver, and submandibular glands, provided protection from deregulation of the oxidative/reductive status in the serum, and decreased the body burden of this toxic metal [12,13,14,15,16,17].

The liver is one of the critical organs for the toxicity of Cd [1,9,10,11,12,13]. The findings of recent epidemiological and experimental data indicate that low chronic intoxication with this xenobiotic may be a cause of damage to this organ [9,10,11,12,13]. We have revealed in our experimental model of human environmental exposure to Cd that treatment with a 1 and 5 mg Cd/kg diet caused pathological changes in the morphological structure of the liver, such as vacuolization and enlarged dimensions of cells, mononuclear cell infiltrations (indicating inflammatory changes), microvascular steatosis, blurred trabecular structure of the lobes, and colliquative necrosis (Table S1) [12]. The injurious impact of this xenobiotic occurred already after three months of low-level intoxication; it worsened with the duration of exposure and depended on its intensity (Table S1) [12]. The mechanism of the injurious action of Cd on the liver is multidirectional, including induction of oxidative stress and lipid peroxidation, development of inflammatory processes, and mitochondrial damage [1,9,12,13]. Moreover, it has been recognized that intoxication with this toxic metal may influence the liver extracellular matrix (ECM), which may have serious consequences for the proper functioning of this organ [18,19,20,21,22,23].

The liver ECM, which constitutes 16–22% of the total volume of the adult human liver, forms a complex and particularly dynamic microenvironment, consisting of various types of macromolecules, which undergoes constant remodeling during development, as well as differentiation and healing due to various lesions [22,23]. Therefore, the proper amount and functionality of the matrix components are necessary to maintain well-coordinated remodeling and homeostasis of this organ [22,23,24]. It has been shown that collagen, which is a key component of the ECM, accounts for over 60% of the matrix of the human liver, including 43% of collagen type I (collagen I) and 12.5% of collagen type III (collagen III) [22]. Collagen provides not only the fibrous scaffold, but it also participates in the transduction of extracellular signals transmitted by integrin receptors present on the cell surface that affect the expression of genes that are critical for cell adhesion, growth, and migration. Collagen III forms heterotypic fibers with collagen I, and their co-expression is associated with the regulation of fiber dimensions, such as their diameter [25,26,27].

Experimental data show that exposure to Cd may disturb collagen metabolism, leading to pathological remodeling of the liver tissue [18,19,20,21]. Cd has been reported to stimulate hepatic stellate cells (HSC) to produce collagen [20,28]. Moreover, this element may influence the activity of HSC and Kupffer cells, which produce enzymes controlling the process of tissue remodeling, such as matrix metalloproteinases (MMPs) and their tissue inhibitors (TIMPs) [20,28,29,30]. MMPs are endopeptidases that have a role in numerous processes (both physiological and pathological) occurring in the body, but, most importantly, they degrade components of the ECM [23,29]. In physiological conditions, the activity of these enzymes is regulated by various factors, such as ROS and cytokines, but mainly by TIMPs [23,29]. Destroying the MMP/TIMP system contributes to dysregulation of homeostasis between the processes of degradation and synthesis of components of the ECM, which may result in the development of pathological states [23,29]. Although MMPs and TIMPs are important for the maintenance of the homeostasis of the liver [23,29], the data on the effect of long-term exposure to Cd on the MMP/TIMP system is very limited; therefore, the involvement of this system in this metal hepatotoxicity is still poorly understood.

Considering our previous findings on the effectiveness of AE in the protection from various unfavorable effects of Cd intoxication, including damage to the liver (Table S1) [12,13,14,15,16,17], as well as the possible mechanisms of the hepatotoxic action of this xenobiotic [1,9,12,13,14,15,16,17,18,19,20,21] and the available—however, limited—data showing that polyphenols or products rich in these compounds are capable of ameliorating Cd-caused destruction in the metabolism of collagen in some tissues [15,18,21,31,32], it was reasonable to hypothesize that the beneficial influence of the extract from chokeberries on the hepatic tissue under exposure to this xenobiotic may also be related to its impact on this main component of the ECM. In the present study, in order to verify this hypothesis, the concentration of total collagen, expression of the most abundant types of this protein found in this organ (collagen I and III) at the messenger ribonucleic acid (mRNA) and protein levels, and the concentrations of matrix metalloproteinase-1 (MMP-1) and matrix metalloproteinase-2 (MMP-2), as well as their tissue inhibitors—tissue inhibitor of metalloproteinase-1 (TIMP-1) and tissue inhibitor of metalloproteinase-2 (TIMP-2)—were assayed in the livers of rats in the experimental model created by us and used in our previous research [12,13,14,15,16,17] that reflects human environmental exposure to Cd. Because it is difficult to identify destruction of the MMP/TIMP balance in the liver in live state, the indices of this system were also determined in the serum, as were correlations between these parameters in the liver and serum, as well as between them and the liver Cd concentration; these were analyzed to recognize their mutual dependencies. Recognition of dependencies between particular indices of the MMP/TIMP balance in the liver and serum may be useful in the early prediction of destruction of collagen homeostasis in this organ. Owing to the fact that balance between particular components of the ECM is of high importance for the proper status of the liver, the ratios of collagen III expression to collagen I expression at the levels of mRNA and protein were calculated. To estimate the MMP/TIMP balance, the ratios of the concentrations of MMP-1 to TIMP-1, MMP-2 to TIMP-2, and MMP-2 to TIMP-1 were calculated as well. Moreover, it was expected that the results of this research would provide a further explanation of the pathways of Cd hepatotoxicity at low exposure. According to our knowledge, a similar investigation has not yet been performed.

## 2. Materials and Methods

### 2.1. Ethical Statement

The experimental model was approved by the Local Ethics Committee for Animal Experiments in Bialystok, Poland (approval No. 60/2009 and 34/2015 issued on 21 September 2009 and 25 March 2015). The study was conducted in accordance with the institutional and international ethical guidelines applicable to animal experiments.

### 2.2. Diets Containing Cd

Feed amended with 1 or 5 mg Cd/kg was prepared by Label Food “Morawski’’ (Kcynia, Poland) by adding, during production, cadmium chloride (CdCl_2_ × 2½ H_2_O; POCh, Gliwice, Poland) to the Labofeed H diet (used in the course of the first 3 months) and standard Labofeed B diet (applied from the 4th month). In our laboratory, the homogeneity of the feed with respect to the content of this element was also checked, and it agreed with the certified values, reaching 1.09 ± 0.13 (mean ± standard deviation) and 4.92 ± 0.53 mg/kg in the 1 and 5 mg Cd/kg diets, respectively [17]. The mean Cd concentration in the standard breeding (Labofeed H diet) and maintenance (Labofeed B diet) diets was <0.06 mg/kg [17].

### 2.3. Extract from the Berries of A. melanocarpa

The extract from the berries of *A. melanocarpa* was received from Adamed Consumer Healthcare (Tuszyn, Poland). According to the manufacturer, the concentrations of total polyphenolic compounds and anthocyanins in the extract reached 65.74% and 18.65%, respectively (Certificate KJ 4/2010) [15,17]. The profile and content of polyphenols in the extract were also investigated in our laboratory. The commercial extract contained 612.40 ± 3.33 mg/g (mean ± standard error–SE) of total polyphenols, 202.28 ± 1.28 mg/g of anthocyanins (80.07 ± 1.05 mg/g—cyanidin 3-*O*-β-galactoside, 33.21 ± 0.01 mg/g—cyanidin 3-*O*-α-arabinoside, and 3.68 ± 0.01 mg/g—cyanidin 3-*O*-β-glucoside), 129.87 ± 1.12 mg/g of proanthocyanidins, 110.92 ± 0.89 mg/g of phenolic acids (68.32 ± 0.08 mg/g—chlorogenic acid), and 21.94 ± 0.98 mg/g of flavonoids [15]. Apart from polyphenols, the chokeberry extract also contained vitamins and minerals, pectins, carotenoids, phytosterols, and triterpenes, as well as sugar and sugar alcohols (producer data). AE was given to rats as a 0.1% aqueous extract (1 g of the lyophilized extract was dissolved in 1 L of redistilled water, and the solution was stable for 24 h after its preparation). The 0.1% AE contained 0.612 ± 0.003 mg/mL (mean ± standard error (SE)) of total polyphenols and <0.05 µg Cd/L [15,17].

### 2.4. Study Design

The study was conducted on 192 female Wistar rats (Crl: WI (Han); certified Laboratory Animal House in Brwinów, Poland) at the age of 3–4 weeks. The rodents were adapted to experimental conditions for 5 days before the beginning of the study. The rats were housed at the temperature of 22 ± 2 °C and relative humidity of 50% ± 10%, were maintained under a 12 h light/dark cycle, and had free access to feed and water during the whole investigation (3, 10, 17, and 24 months). The animals were divided (randomly) into six groups (32 individuals per group) as follows:

- Control: Received water without the AE addition (<0.05 μg Cd/L) and the standard feed.
- AE: Received the 0.1% aqueous solution of AE as the only drinking fluid.
- Cd_1_: Intoxicated with Cd at the concentration of 1 mg/kg diet.
- Cd_1_ + AE: Received the 0.1% AE and the feed containing 1 mg Cd/kg.
- Cd_5_: Treated with Cd at the concentration of 5 mg/kg diet.
- Cd_5_ + AE: Received the 0.1% AE and the feed containing 5 mg Cd/kg.

The average daily Cd intake by the rats during the 24-month experiment was 37.50–84.88 µg/kg body weight (b.w.) in the Cd_1_ and Cd_1_ + AE groups and 196.69–404.76 µg/kg b.w. in the Cd_5_ and Cd_5_ + AE groups. The mean daily consumption of AE ranged between 63.1 and 159.1 mg/kg b.w. (polyphenol intake was 41.5–104.6 mg/kg b.w.) [17]. The mean intake of Cd and the extract at particular time points did not differ depending on the manner of their administration (together or separately) [17]. Through the whole study, no differences in the consumption of drinking water and food or in the weight gain were noted among the particular groups of rats [17]. The evaluation of Cd concentration in the urine and blood of the animals maintained on the 1 and 5 mg Cd/kg diets alone or together with AE (0.085–0.354 μg/g creatinine and 0.103–0.324 μg/L, and 0.284–0.820 μg/g creatinine and 0.584–1.332 μg/L, respectively) [17] confirmed that the experimental model used in this study well reflects environmental exposure to this xenobiotic in economically developed areas [8,9,10,11].

During the experiment, no signs of morbidity and mortality were noted, except for 3 unprompted deaths between the 17th and 24th month (one case in each of the AE, Cd_1_, and Cd_5_ groups).

At the end of the study (consecutively after 3, 10, 17, and 24 months), the rats were sacrificed under intraperitoneal barbiturate anesthesia (Morbital—30 mg/kg b.w.; Biowet, Pulawy, Poland). All of the blood was collected by cardiac puncture with or without anticoagulant (heparin; Biochemie GmbH, Kundl, Austria), and the liver and other organs were removed and rinsed with cold physiological saline. The biological material, including the liver used in the current study, was kept at −70 °C until the assay.

The experimental model used here has been reported in detail elsewhere [12,13,14,15,16,17].

### 2.5. Assay of the Concentration of Total Collagen in the Liver

The slices of the hepatic tissue (about 100 mg, always from the same liver lobe) were homogenized in 1 mL of 50 mM Tris-HCl cold buffer, pH = 7.5 (GenoPlast Biochemicals, Rokocin, Poland) containing 0.2 M sodium chloride (NaCl; Eurochem BGD, Tarnów, Polska) and inhibitors of proteinases—5 mM ethylenediaminetetraacetic acid (Avantor Performance Materials Poland SA, Gliwice, Poland), 5 mM phenylmethylsulfonyl fluoride (Sigma-Aldrich, Saint Louis, MO, USA), and 10 mM *N*-ethylmaleimide (Sigma-Aldrich)—using a high-performance homogenizer (Ultra-Turrax T25, IKA, Staufen, Germany). The homogenates were centrifuged (Centrifuge 5415R, Eppendorf, Hamburg, Germany) at 16,000 × *g* for 15 min (4 °C), and the separated aliquots were kept at −70 °C until the measurements of the concentrations of collagen and total non-collagen protein were performed.

The concentration of total soluble collagen in the aliquots of the liver homogenates was assessed by the Sircol^TM^ Soluble Collagen Assay (Biocolor Ltd., County Antrim, U.K.) in accordance with the instructions of the manufacturer. Briefly, Sircol Dye Reagent (1 mL) was added to 100 µL of the assayed sample, shaken with the use of an orbital shaker (Thermomixer comfort, Hamburg, Germany) for 30 min at room temperature, and then centrifuged at 9300× *g* for 10 min at room temperature. The pellet of the collagen–dye complex was rinsed of unbound dye with ice-cold Acid-Salt Wash Reagent, and after centrifugation (9300× *g* for 10 min at room temperature), it was suspended in 250 µL of Alkali Reagent containing 0.5 M sodium hydroxide. The absorbance of the samples was recorded at 555 nm using a microplate reader (Infinite M200, Tecan, Männedorf, Switzerland). The concentration of total collagen was read from the standard curve made from the standard of collagen I provided with the assay kit. Moreover, the concentration of total non-collagen protein was measured in the aliquots with the use of the BCA Protein Assay Kit (Pierce, Rockford, IL, USA). The concentration of total collagen in the liver was normalized to the total non-collagen protein.

### 2.6. Determination of the Expression of Collagen I and III at the mRNA Level in the Liver

#### 2.6.1. Isolation of Total Ribonucleic Acid (RNA) from the Liver Tissue

For isolation of total RNA, the liver tissue (50–100 mg) was homogenized in 1 mL of TRIsure™ Reagent (Bioline GmbH, Luckenwalde, Germany) with the use of a high-performance homogenizer (Ultra-Turrax T25, IKA, Staufen, Germany). Lysates were centrifuged (Centrifuge 5415R, Eppendorf, Hamburg, Germany) at 12,000× *g* for 5 min (4 °C) and then incubated for 5 min for complete dissociation of the complex of nucleoproteins. After that time, 0.2 mL of chloroform (Sigma-Aldrich) was added to the samples and shaken vigorously (Thermomixer comfort, Hamburg, Germany) for 15 s, and was left for 3 min at room temperature. Then, the samples were centrifuged for 15 min at 12,000× *g* (4 °C), and the resulting RNA-containing aqueous phase was transferred to new test tubes. RNA was precipitated with isopropyl alcohol (Sigma-Aldrich) by adding 0.5 mL to the sample and leaving the sample for 10 min at room temperature, followed by centrifugation at 12,000× *g* for 10 min (4 °C). The obtained pellets were washed once with 1 mL of 75% ethyl alcohol (Sigma-Aldrich) by mixing the samples and underwent centrifugation at 7500× *g* for 5 min (4 °C). After air-drying, the RNA pellet was dissolved in 50 μL of diethyl-pyrocarbonate-treated water (A&A Biotechnology, Gdynia, Poland). The concentration and the degree of RNA purification from proteins (A260nm/A280nm) and organic reagents (A260nm/230nm) were determined using a NanoDrop 2000 Spectrophotometer (Thermo Fisher Scientific, Wilmington, DE, USA). Solutions of RNA were stored at −70 °C.

#### 2.6.2. Synthesis of cDNA and Quantitative Real-Time Polymerase Chain Reaction (Real-Time PCR)

For the synthesis of cDNA, a SensiFAST^TM^ cDNA Synthesis Kit (Bioline, London, UK) was used. The 20 μL reaction mixtures containing equal amounts of total RNA (1 μg) were incubated successively at 25 °C for 10 min, 45 °C for 15 min, and 85 °C for 5 min in a thermal cycler (Thermocycler CFX96 Real-Time System, Bio-Rad, Hercules, CA, USA). The samples of cDNA were stored at −20 °C.

A Real-Time PCR was conducted with the use of a SensiFAST™ SYBR Kit (Bioline). The reaction mixtures contained 2 μL of cDNA (diluted 3 times), 5 μL of SensiFAST™ reagent, and 0.4 μL of forward and reverse primers with concentrations of 10 μM each (Genomed, Warsaw, Poland), and were made up with nuclease-free water to a volume of 10 μL. The sequences of primers used for amplification of the gene *COL1A1* encoding the α1 subunit of collagen I, the gene *COL3A1* encoding collagen III, and the gene *GAPDH* encoding glyceraldehyde-3-phosphate dehydrogenase were as follows: *COL1A*, forward: 5′-ATC AGC CCA AAC CCC AAG GAG A-3′ and reverse: 5′-CGC AGG AAG GTC AGC TGG ATA G-3′; *COL3A1*, forward: 5′-TGA TGG GAT CCA ATG AGG GAG A-3′ and reverse: 5′-GAG TCT CAT GGC CTT GCG TGT TT-3′; *GAPDH*, forward: 5′-CCA TTC TTC CAC CTT TGA TGC T-3′ and reverse: 5′-TGT TGC TGT AGC CAT ATT CAT TGT-3′ [33]. The reaction was performed in a thermocycler (Thermocycler CFX96 Real-Time System, Bio-Rad) using the following amplification parameters: 95 °C for 10 s, 60 °C for 15 s, and 72 °C for 20 s. The quality of PCR products was analyzed on the basis of melting curves obtained by gradually increasing the temperature of the tested mixture in the range of 55–95 °C. The expression of collagen genes was normalized to the *GAPDH* gene and expressed as a fold of changes versus (vs.) untreated controls.

### 2.7. Determination of Collagen I and III Expression at the Protein Level in the Liver 

The liver tissue (50 mg) was homogenized at 4 °C in a 0.5 mL radioimmunoprecipitation assay (RIPA) buffer (Sigma-Aldrich) with added protease inhibitor cocktail (Sigma-Aldrich). After centrifugation at 14,000× *g* for 10 min (4 °C), the concentration of protein was measured in the supernatants using the BCA Protein Assay Kit (Pierce, Rockford, IL, USA). The aliquots of the liver homogenates were stored at −70 °C. 

Samples containing equal amounts of proteins (20 μg) were diluted 1:2 in 2× concentrated 0.125 M Tris-HCl, pH = 6.8 buffer (GenoPlast) containing 4% *w/v* sodium dodecyl sulfate (Sigma-Aldrich), 10% *v*/*v* 2-mercaptoethanol (Sigma-Aldrich), 20% *v*/*v* glycerol (Avantor Performance Materials Poland SA, Gliwice, Poland), and 0.2% *w/v* bromophenol blue (Avantor Performance Materials, Poland SA); after heating (Thermomixer comfort, Hamburg, Germany) at 85 °C for 10 min, they were loaded on 7.5% *w/v* polyacrylamide gel (Sigma-Aldrich). After electrophoresis with the use of Mini-Protean 3 Cell (Bio-Rad, Hercules, CA, USA), proteins from the gels were immobilized by transfer to Immobilon P (Millipore, Billerica, Massachusetts, USA) with the use of Trans-Blot (Bio-Rad). To block any non-specific proteins, 5% (*w/v*) non-fat dry milk (Bio-Rad) dissolved in Tris-buffered saline with Tween (TBS-T) with a composition of 10 mM Tris-HCl, pH = 7.5 (GenoPlast Biochemicals), 150 mM NaCl (Eurochem BGD, Tarnów, Polska), and 0.1% (*v*/*v*) Tween 20 (Sigma-Aldrich) was added to the membranes, and they were stirred at room temperature for 1 h. The membranes were then incubated overnight at 4 °C with primary monoclonal antibodies: anti-collagen I (Santa Cruz Biotechnology Inc., Santa Cruz, CA, USA), anti-collagen III (Abcam, Cambridge, UK), and anti-β-actin (Santa Cruz Biotechnology Inc.), diluted 1:600, 1:500, and 1:3000, respectively, in TBS-T supplemented with 1% (*w/v*) bovine serum albumin (Sigma-Aldrich). The membranes were rinsed (3 × 10 min) using the TBS-T solution. Successively, the membranes were exposed to the anti-mouse immunoglobulin G antibody (at 1:5000 dilution) conjugated with horseradish peroxidase (Santa Cruz Biotechnology Inc.) for 1 h at room temperature and washed again with the TBS-T solution, this time 5 times for 5 min per wash. The signals were detected by enhanced chemiluminescence using Westar Supernova Chemiluminescent Substrate (Cyanagen, Bologna, Italy). For documentation of results, an apparatus for gel documentation (G:BOX, Syngene, Cambridge, UK) was used. Densitometric measurements of the intensity of protein bands were performed using the Gene Tools (Syngene) program. The intensity of the measurements of the collagen bands was normalized to β-actin and expressed as a percentage of untreated controls.

### 2.8. Assessment of the Concentrations of MMPs and TIMPs in the Liver and Serum

#### 2.8.1. Preparation of the Aliquots of the Hepatic Tissue

Ten percentage homogenates of the liver (*w/v*) were prepared by using a high-performance homogenizer (Ultra-Turrax T25, IKA). Known-weight hepatic tissue slices (about 100 mg) were homogenized in 50 mM cold potassium phosphate buffer (pH = 7.4). The buffer was made by mixing 1 M potassium dihydrogen phosphate (POCh), 1 M dipotassium hydrogen phosphate (POCh), and distilled water. After the centrifugation of the homogenates (MPW-350R centrifuge, Medical Instruments, Warsaw, Poland) at 700× *g* for 20 min at 4 °C, the aliquots were immediately separated and stored at −70 °C.

#### 2.8.2. Assay of MMPs and TIMPs in the Aliquots of the Liver Tissue and in the Serum

The concentrations of MMPs and TIMPs were determined using the specific Rat(MMP-1), Rat(MMP-2), Rat(TIMP-1), and Rat(TIMP-2) double-antibody sandwich enzyme-linked immunosorbent assay (ELISA) kits purchased from SunRed (Shanghai, China) in accordance with the manufacturers’ instructions. The intra- and inter-assay coefficients of variation (CV) were <4% and <4%, respectively, for MMP-1, <4% and <3% for MMP-2, <3% and <5% for TIMP-1, and <3% and <3.5% for TIMP-2 in the liver, as well as <4.3% and <3% for MMP-1, <3% and <2.5% for MMP-2, <4.7% and <4% for TIMP-1, and <1.9% and <2% for TIMP-2 in the serum.

All measured parameters were adjusted for the concentration of total protein assayed using the Total Protein BioMaxima Kit (Lublin, Poland) (intra-assay CV <2.2%).

### 2.9. Statistical Analysis

The results were statistically analyzed using the Statistica 12 software (StatSoft, Tulsa, OK, USA). Numerical data are expressed as mean ± SE. A nonparametric signed-rank Kruskal–Wallis test was performed to recognize whether statistically significant differences existed among the experimental groups (*p* < 0.05). A Kruskal–Wallis post hoc test was carried out to disclose the occurrence of difference (*p* < 0.05) between two means. In figures and tables, statistically significant differences in relation to the control group, the respective groups receiving Cd or AE alone, and the respective groups exposed to the 1 mg Cd/kg diet alone or with AE are marked.

In the situation of revealing (Kruskal–Wallis test) any impact of the simultaneous administration of Cd and AE on the measured parameter, a two-way analysis of variance (ANOVA/MANOVA) was carried out in order to disclose the main effects of these agents and the interaction between them (test *F*, *p* < 0.05). In the case when the interactive effect of Cd and AE was found, the potential nature of this interaction was estimated. For this purpose, the effect of co-administration of this xenobiotic and the extract was compared to the sum of effects recorded when these agents were used separately. These effects were expressed as percentage changes (in cases where the change was less than 100%) or a factor of change (in cases where the change reached or exceeded 100%) of an assayed variable in comparison to the value of this parameter in the control animals. Such calculations allow one to estimate if the interaction is additive (Cd + AE effect = Cd effect + AE effect), antagonistic (Cd + AE effect < Cd effect + AE effect), or synergistic (Cd + AE effect > Cd effect + AE effect) in character [13].

Mutual correlations (Spearman rank correlation analysis) among the assayed parameters, as well as between these parameters and the Cd concentration in the liver previously determined by us [17], were also determined. A correlation is considered statistically significant in the case when a correlation coefficient (*r*) has *p* < 0.05.

## 3. Results

### 3.1. The Concentration of Total Collagen in the Liver

**Figure 1:**
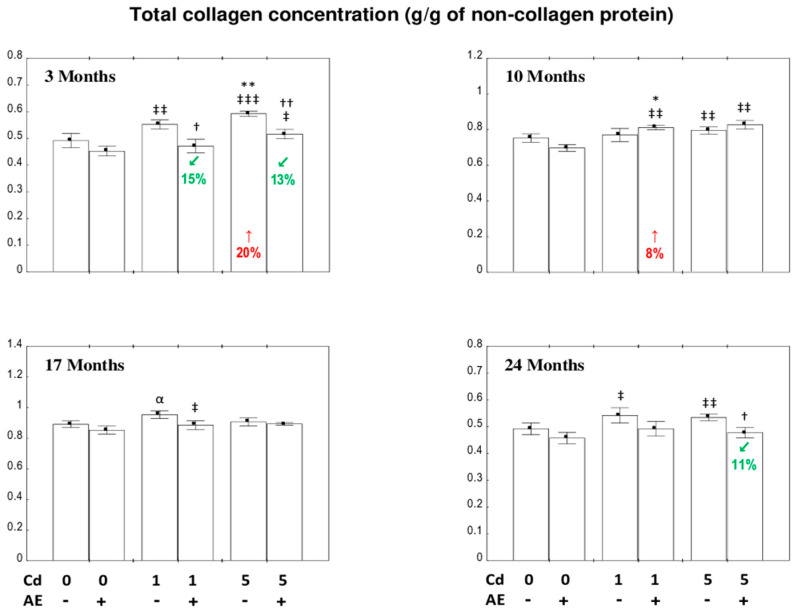
The impact of extract from *Aronia melanocarpa* L. berries (AE) on the concentration of total collagen in the livers of rats exposed to cadmium (Cd). The animals were treated with Cd at the concentrations of 0, 1, and 5 mg Cd/kg in their diets and/or 0.1% aqueous AE (+) or not (−). Data are shown as mean ± standard error (SE) for eight rats, except for seven animals in the AE, Cd_1_, and Cd_5_ groups after 24 months. Statistically significant differences (Kruskal–Wallis post hoc test): * p < 0.05, ** *p* < 0.01, and ^α^ *p* < 0.06 vs. control group; ^†^ *p* < 0.05 and ^††^ *p* < 0.01 vs. appropriate Cd group; ^‡^ *p* < 0.05, ^‡‡^ *p* < 0.01, and ^‡‡‡^ *p* < 0.001 vs. AE group. Numerical values in bars express the percentage changes in comparison to the control group (↑, increase) or the appropriate group exposed to Cd alone (↙, decrease).

The consumption of AE or Cd alone did not affect the concentration of total collagen during the whole experimental period, except for an increase (by 20%) in this parameter in the group fed for three months with the 5 mg Cd/kg diet and a visible tendency (*p* < 0.06) to its increase after intoxication with the 1 mg Cd/kg diet for 17 months (Figure 1). The administration of chokeberry extract to the animals intoxicated with this xenobiotic resulted in a decrease in the concentration of total collagen in the Cd_1_ + AE and Cd_5_ + AE groups (by 15% and 13%, respectively) compared to the respective Cd groups (Cd_1_ or Cd_5_) after three months, as well as in the Cd_5_ + AE group (by 11%) in comparison to the Cd_5_ group after 24 months of the experiment (Figure 1). The two-way analysis of variance showed that the impact of the consumption of the extract from chokeberries was the result of the independent action of its components (*F* = 5.885–9.716, *p* < 0.05–0.01) (Table S2). Moreover, a rise in the level of total collagen in the Cd_1_ + AE group vs. the control group after 10 months of the investigation was noted; however, as was revealed based on the ANOVA/MANOVA analysis, this change was solely an effect of the independent influence of Cd (*F* = 7.277, *p* < 0.05) (Table S2).

### 3.2. The Expression of Collagen I and III at the mRNA Level in the Liver

**Figure 2:**
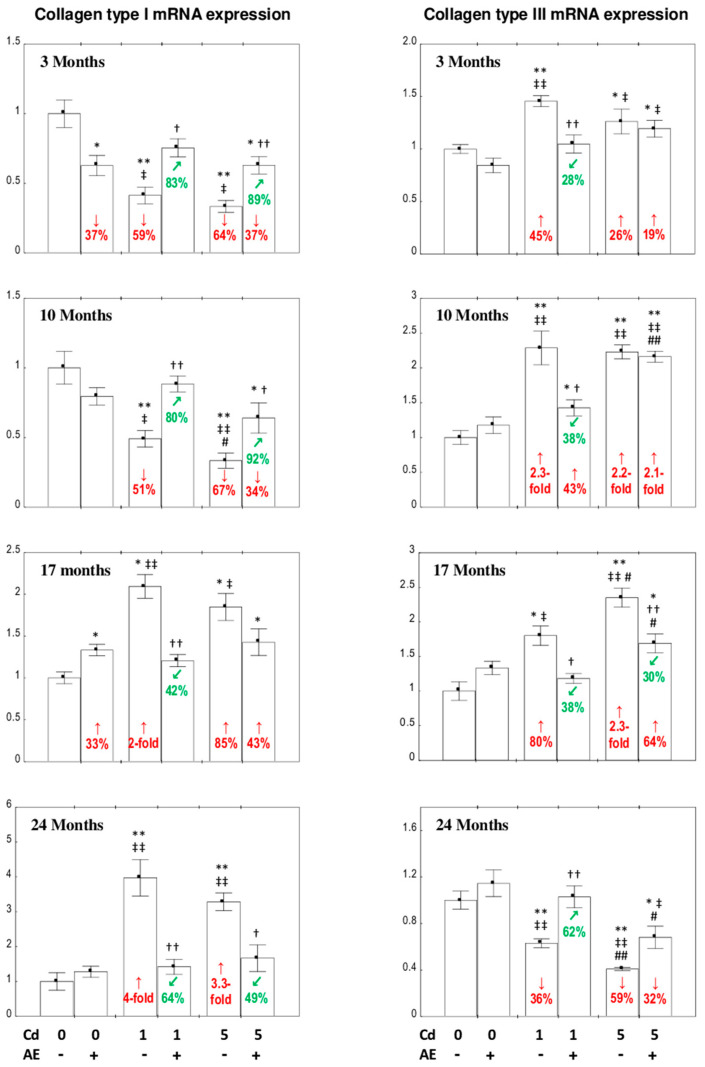
The impact of *Aronia melanocarpa* L. berry extract (AE) on the expression of collagen type I and collagen type III at the messenger ribonucleic acid (mRNA) level in the livers of rats exposed to cadmium (Cd). The animals were treated with Cd at the concentrations of 0, 1, and 5 mg Cd/kg in their diets and/or 0.1% aqueous AE (+) or not (−). Data are shown as mean ± SE for eight rats, except for seven animals in the AE, Cd_1_, and Cd_5_ groups after 24 months. Statistically significant differences (Kruskal–Wallis post hoc test): * p < 0.05 and ** *p* < 0.01 vs. control group; ^†^ *p* < 0.05 and ^††^ *p* < 0.01 vs. appropriate Cd group; ^‡^ *p* < 0.05 and ^‡‡^ *p* < 0.01 vs. AE group; ^#^ *p* < 0.05 and ^##^ *p* < 0.01 vs. appropriate group maintained on the 1 mg Cd/kg diet (Cd_1_ group or Cd_1_ + AE group). Numerical values in bars express the percentage changes or factors of changes in comparison to the control group (↑, increase; ↓, decrease) or the appropriate group exposed to Cd alone (↗, increase; ↙, decrease).

The intake of AE alone throughout the 24-month experiment had no influence on the expression of the *COL1A1* gene encoding collagen type I (collagen I mRNA) or the *COL3A1* gene encoding collagen type III (collagen III mRNA), apart from a reduction in the expression of collagen I mRNA and its increase after 3 and 17 months of the investigation, respectively (Figure 2).

In the rats fed with the 1 and 5 mg Cd/kg diets, the expression of collagen I mRNA was decreased (by 51–67%) after 3 and 10 months, and increased (by 85% to four-fold) after 17 and 24 months of the experiment (Figure 2). The application of AE in the case of Cd intoxication (entirely or partially) prevented these metal-induced changes in the expression of this protein at the mRNA level, except in the Cd_5_ + AE group after 17 months (Figure 2). Although the expression of collagen I mRNA in the Cd_5_ + AE group at this time point was within the range of the Cd_5_ group, it was higher by 43% than in the control group, whereas the increase in the Cd_5_ group reached 85% (Figure 2). Moreover, the two-way analysis of variance confirmed the effect of AE administration on the expression of collagen I mRNA in the Cd_5_ + AE group after 17 months (interactive effect of the extract ingredients and Cd; *F* = 9.321, *p* < 0.01; Table 1). The exposure to Cd increased (by 26% to 2.3-fold) the expression of collagen III mRNA for up to 17 months, and decreased its expression after 24 months of the study (by 36% and 59% in the Cd_1_ and Cd_5_ groups, respectively) (Figure 2). The intake of AE during the low exposure to this xenobiotic entirely or partially prevented the influence of Cd on collagen III mRNA expression, while under moderate intoxication, the extract offered only partial protection against the 17-month impact of this metal (Figure 2). There was no difference in the expression of collagen III mRNA in the Cd_5_ and Cd_5_ + AE groups after 24 months; however, in the first group, the expression was lower by 59% compared to the control, whereas in the second group, the decrease (by 32%) was clearly smaller (Figure 2). Moreover, the two-way analysis of variance confirmed the effect of AE (*F* = 6.196, *p* < 0.05) on collagen III expression (Table 1). Generally, the influence of the chokeberry extract on the expression of collagen I and III mRNA in the hepatic tissue of animals intoxicated with Cd resulted from the independent action of its components (*F* = 5.898–12.69, *p* < 0.05–0.01 and *F* = 4.851–18.81, *p* < 0.05–0.001, respectively) and/or their interaction with this xenobiotic (*F* = 8.056–42.48, *p* < 0.05–0.001 and *F* = 11.26–17.69, *p* < 0.01–0.001, respectively), which was antagonistic in character (Table 1). According to the ANOVA/MANOVA analysis, the enhancement of the expression of collagen III mRNA in the Cd_5_ + AE group after 3 and 10 months was the main effect of Cd action (*F* = 13.81, *p* < 0.01 and *F* = 122.5, *p* < 0.001, respectively) and was not caused by the administration of AE (Table 1).

No differences in the expression of collagen I mRNA in the liver tissue were disclosed between the appropriate groups administered with the 1 or 5 mg Cd/kg diet alone or with AE, except for its lower (by 32%) expression at the moderate-level (Cd_5_ group) than at the low-level (Cd_1_ group) exposure for 10 months (Figure 2). The expression of collagen III mRNA in the rats treated with this toxic metal alone for 17 and 24 months and together with AE for 10, 17, and 24 months was dependent on the intensity of the intoxication (Figure 2).

### 3.3. The Expression of Collagen I and III at the Protein Level in the Liver

**Figure 3:**
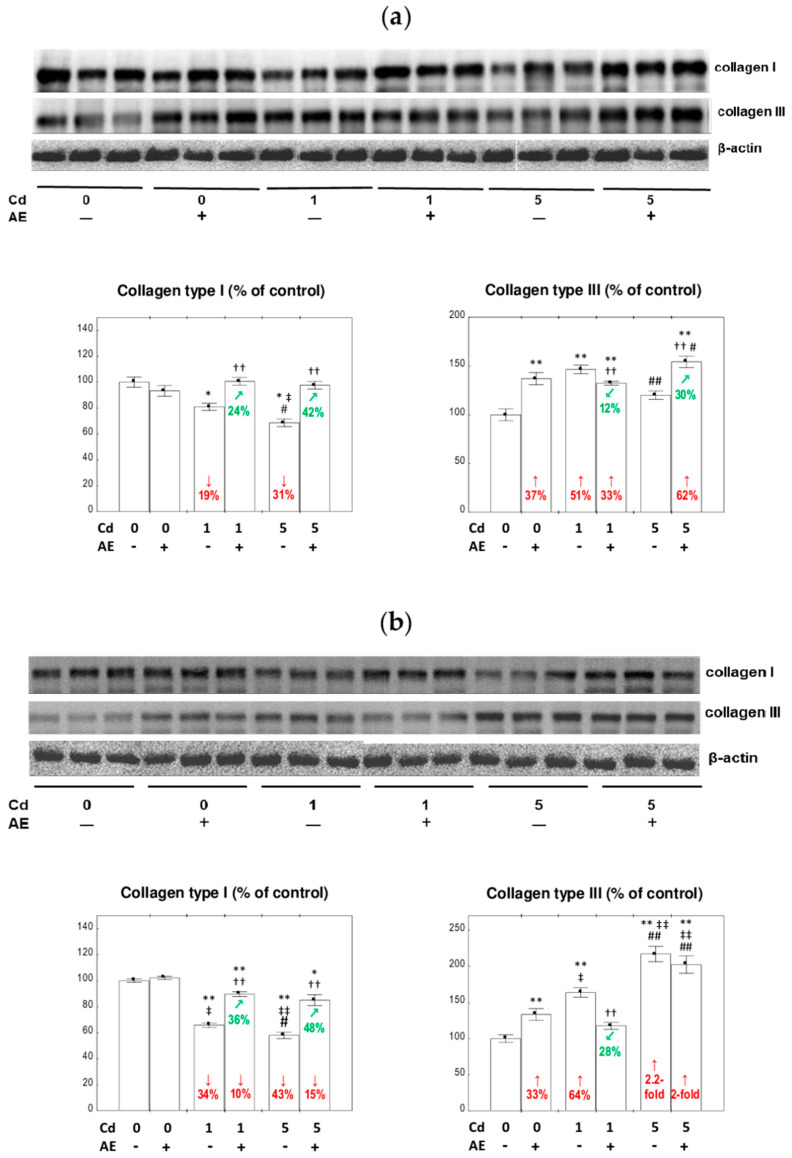
The impact of *Aronia melanocarpa* L. berry extract (AE) on the expression of collagen type I and collagen type III at the protein level in the livers of rats exposed to cadmium (Cd) for 3 (**a**) and 10 (**b**) months. The animals were treated with Cd at the concentrations of 0, 1, and 5 mg Cd/kg in their diets and/or 0.1% aqueous AE (+) or not (−). Representative gels of Western blotting are presented. Data are shown as mean ± SE for eight rats. Statistically significant differences (Kruskal–Wallis post hoc test): * p < 0.05 and ** *p* < 0.01 vs. control group; ^††^ *p* < 0.01 vs. appropriate Cd group; ^‡^ *p* < 0.05 and ^‡‡^ *p* < 0.01 vs. AE group; ^#^ *p* < 0.05 and ^##^ *p* < 0.01 vs. appropriate group maintained on the 1 mg Cd/kg diet (Cd_1_ group or Cd_1_ + AE group). Numerical values in bars express the percentage changes or factors of changes in comparison to the control group (↑, increase; ↓, decrease) or the appropriate group exposed to Cd alone (↗, increase; ↙, decrease).

**Figure 4:**
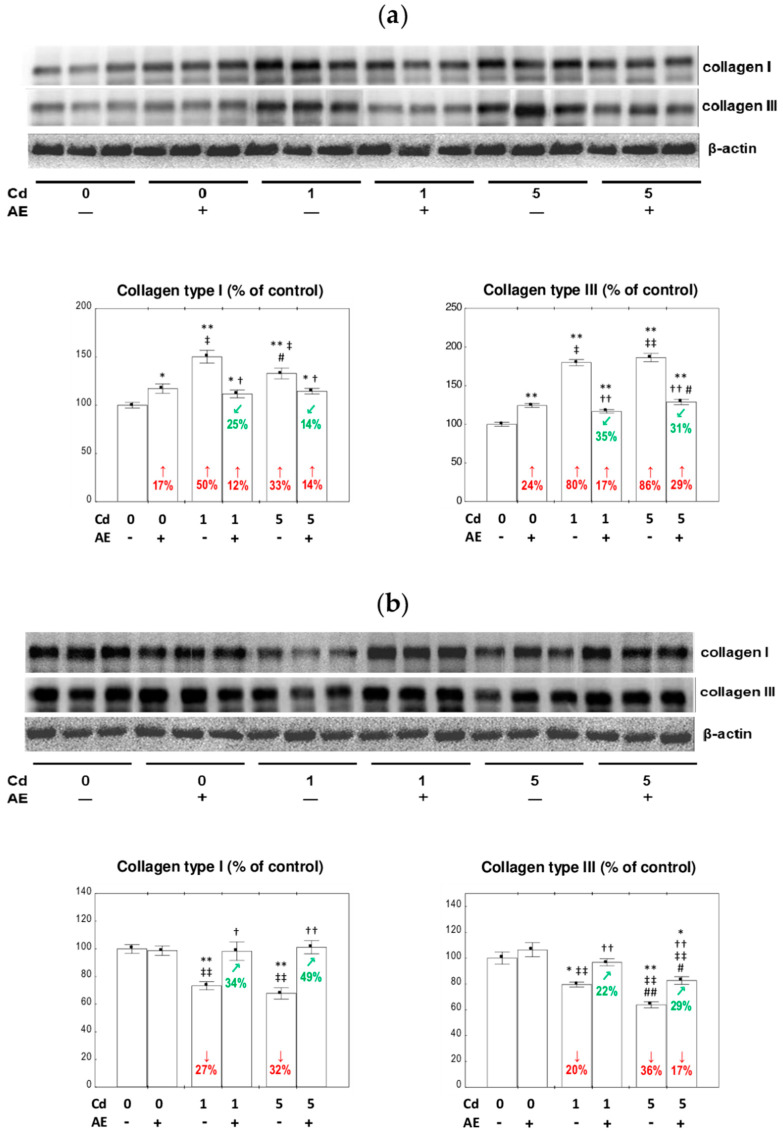
The impact of *Aronia melanocarpa* L. berry extract (AE) on the expression of collagen type I and collagen type III at the protein level in the livers of rats exposed to cadmium (Cd) for 17 (**a**) and 24 (**b**) months. The animals were treated with Cd at the concentrations of 0, 1, and 5 mg Cd/kg in their diets and/or 0.1% aqueous AE (+) or not (−). Representative gels of Western blotting are presented. Data are shown as mean ± SE for eight rats, except for seven animals in the AE, Cd_1_, and Cd_5_ groups after 24 months. Statistically significant differences (Kruskal–Wallis post hoc test): * p < 0.05 and ** *p* < 0.01 vs. control group; ^†^ *p* < 0.05 and ^††^ *p* < 0.01 vs. appropriate Cd group; ^‡^ *p* < 0.05 and ^‡‡^ *p* < 0.01 vs. AE group; ^#^ *p* < 0.05 and ^##^ *p* < 0.01 vs. appropriate group maintained on the 1 mg Cd/kg diet (Cd_1_ group or Cd_1_ + AE group). Numerical values in bars express the percentage changes or factors of changes in comparison to the control group (↑, increase; ↓, decrease) or the appropriate group exposed to Cd alone (↗, increase; ↙, decrease)

The supplementation with AE alone did not influence the expression of collagen I at the protein level in the liver, except for an increase after 17 months, whereas collagen III expression was enhanced for the first 17 months, and after 24 months, it was within the range of the values noted in the control animals (Figure 3 and Figure 4).

The treatment with the 1 and 5 mg Cd/kg diets resulted in a decrease (by 19–43%) in collagen I expression at the protein level after 3, 10, and 24 months and in its increase (by 50% and 33%, respectively) after 17 months. Both low and moderate exposure to this xenobiotic increased (by 51% to 2.2-fold) the expression of collagen III after 3–17 months, except for the Cd_5_ group after three months, and decreased (by 20% and 36%, respectively) the expression after 24 months (Figure 3 and Figure 4). The supplementation with AE along with the feeding with the 1 and 5 mg Cd/kg diets entirely or partially protected from Cd-induced alterations in collagen I and III expression at the protein level, except for a lack of impact on collagen III expression after 10 months of the moderate exposure, which remained increased vs. the control group (Figure 3 and Figure 4). The beneficial impact of AE on the expression of collagen I and III at the protein level in the livers of rats treated with Cd resulted from the independent action of the extract (*F* = 4.297–72.18, *p* < 0.05–0.001 and *F* = 5.407–43.15, *p* < 0.05–0.001, respectively) and/or its antagonistic interaction with this xenobiotic (*F* = 9.121–50.73, *p* < 0.01–0.001 and *F* = 6.636–217.8, *p* < 0.05–0.001, respectively) (Table 2). The consumption of AE by the animals fed with the 5 mg Cd/kg diet for three months increased the expression of collagen III at the protein level with respect to the control group, and this effect resulted from the independent action of the extract ingredients (*F* = 39.33, *p* < 0.001) (Table 2).

After 3, 10, and 17 months of the experiment, significant differences in collagen I expression dependent on the level of exposure to Cd alone were observed (Figure 3 and Figure 4). No differences were noted in the expression of this protein between the Cd_1_ + AE and Cd_5_ + AE groups (Figure 3 and Figure 4). The expression of collagen III clearly depended on the intensity of intoxication with Cd in all experimental groups, apart from the 17-month low-level and moderate treatment (Figure 3 and Figure 4). 

### 3.4. The Ratio of the Expression of Collagen III to Collagen I in the Liver

**Figure 5:**
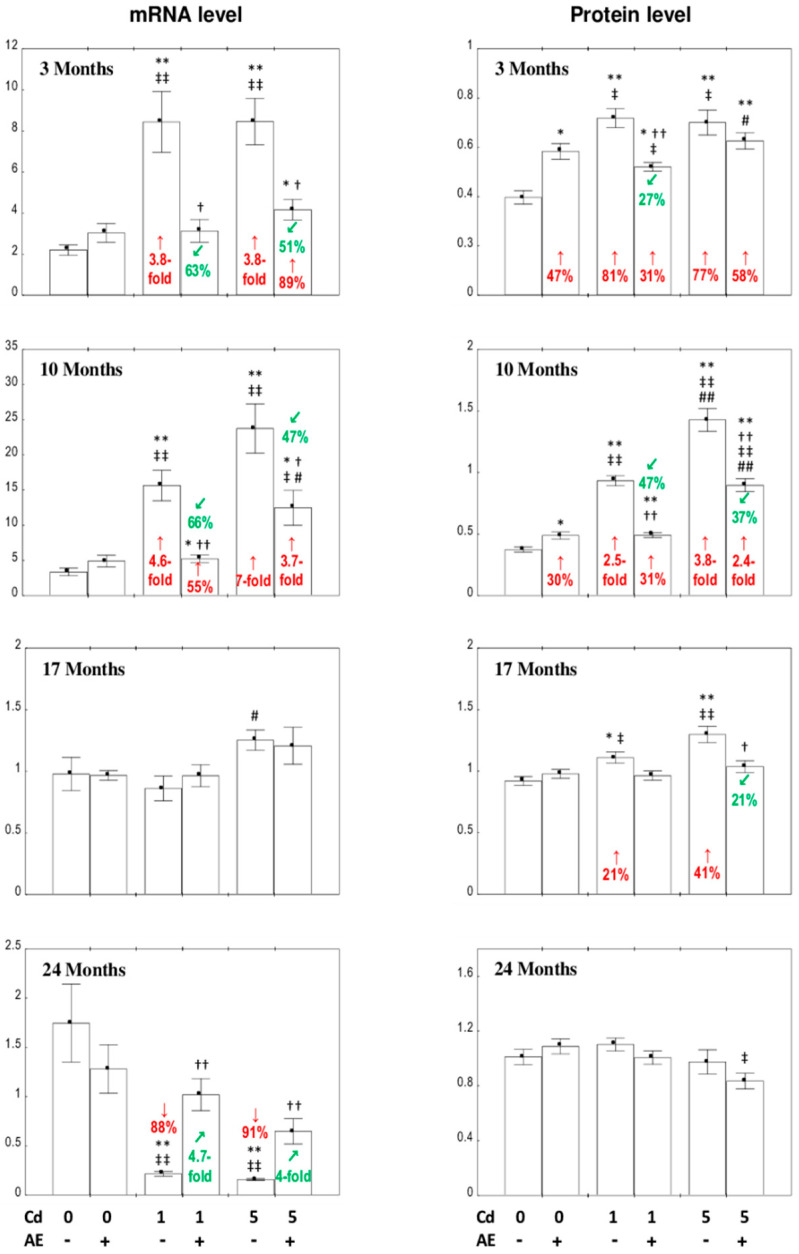
The impact of *Aronia melanocarpa* L. berry extract (AE) on the ratio of the expression of collagen type III to that of collagen type I at the mRNA and protein levels in the livers of rats exposed to cadmium (Cd). The animals were treated with Cd at the concentrations of 0, 1, and 5 mg Cd/kg in their diets and/or 0.1% aqueous AE (+) or not (−). Data are shown as mean ± SE for eight rats, except for seven animals in the AE, Cd_1_, and Cd_5_ groups after 24 months. Statistically significant differences (Kruskal–Wallis post hoc test): * p < 0.05 and ** *p* < 0.01 vs. control group; ^†^ *p* < 0.05 and ^††^ *p* < 0.01 vs. appropriate Cd group; ^‡^ *p* < 0.05 and ^‡‡^ *p* < 0.01 vs. AE group; ^#^ *p* < 0.05 and ^##^ *p* < 0.01 vs. appropriate group maintained on the 1 mg Cd/kg diet (Cd_1_ group or Cd_1_ + AE group). Numerical values in bars (or above the bars) express the percentage changes or factors of changes in comparison to the control group (↑, increase; ↓, decrease) or the appropriate group exposed to Cd alone (↗, increase; ↙, decrease).

The intake of AE alone during the experiment did not change the ratio of the expression of collagen III to collagen I at the levels of mRNA and protein in the liver, apart from an increase in this ratio at the protein level after 3 and 10 months (by 47% and 30%, respectively) (Figure 5).

The dietary exposure to Cd at the concentrations of 1 and 5 mg/kg led to an increase in the ratio of the expression of collagen III to that of collagen I at the levels of mRNA and protein after 3 and 10 months (by 77% to seven-fold) and at the protein level after 17 months of the study (by 21% and 41%, respectively) (Figure 5). The intoxication with Cd for 24 months caused a decrease in this ratio only at the mRNA level (Figure 5). The administration of AE to the animals intoxicated with Cd offered protection (total or partial) against this xenobiotic influence on the ratio of the expression of collagen III to that of collagen I at both levels, except for the ratio at the protein level after three months of being maintained on the diet containing 5 mg Cd/kg (Figure 5). The beneficial impact of the extract on the collagen III/collagen I expression ratio in the liver resulted from the independent action of AE (*F* = 4.459–32.18, *p* < 0.05–0.001) (Table 3) and/or an antagonistic interactive action of its ingredients with Cd (*F* = 6.586–92.83, *p* < 0.05–0.001) (Table 3). The analysis revealed no independent impact of AE and/or its interactive action with Cd (Table 3) on the ratio of the expression of collagen III to collagen I at the mRNA level after 24 months of exposure to the 5 mg Cd/kg diet despite the favorable impact of its administration recognized by the Kruskal–Wallis post hoc test (Figure 5).

The ratio of the expression of collagen III to that of collagen I in the liver depending on the intensity of intoxication with this heavy metal had a higher value in the Cd_5_ + AE group (by 4.4-fold) than in the Cd_1_ + AE group, as well as in the Cd_5_ group (by 45%) compared to the Cd_1_ group, at the mRNA level after 10 and 17 months, respectively; a higher value of the ratio was found for the Cd_5_ + AE group compared to the Cd_1_ + AE group after 3 and 10 months (by 20% and 82%, respectively), and for the Cd_5_ group (by 53%) vs. the Cd_1_ group at the protein level after 10 months of the investigation (Figure 5).

### 3.5. The Concentrations of MMPs and TIMPs in the Liver

#### 3.5.1. The Concentrations of MMP-1 and MMP-2 in the Liver

**Figure 6:**
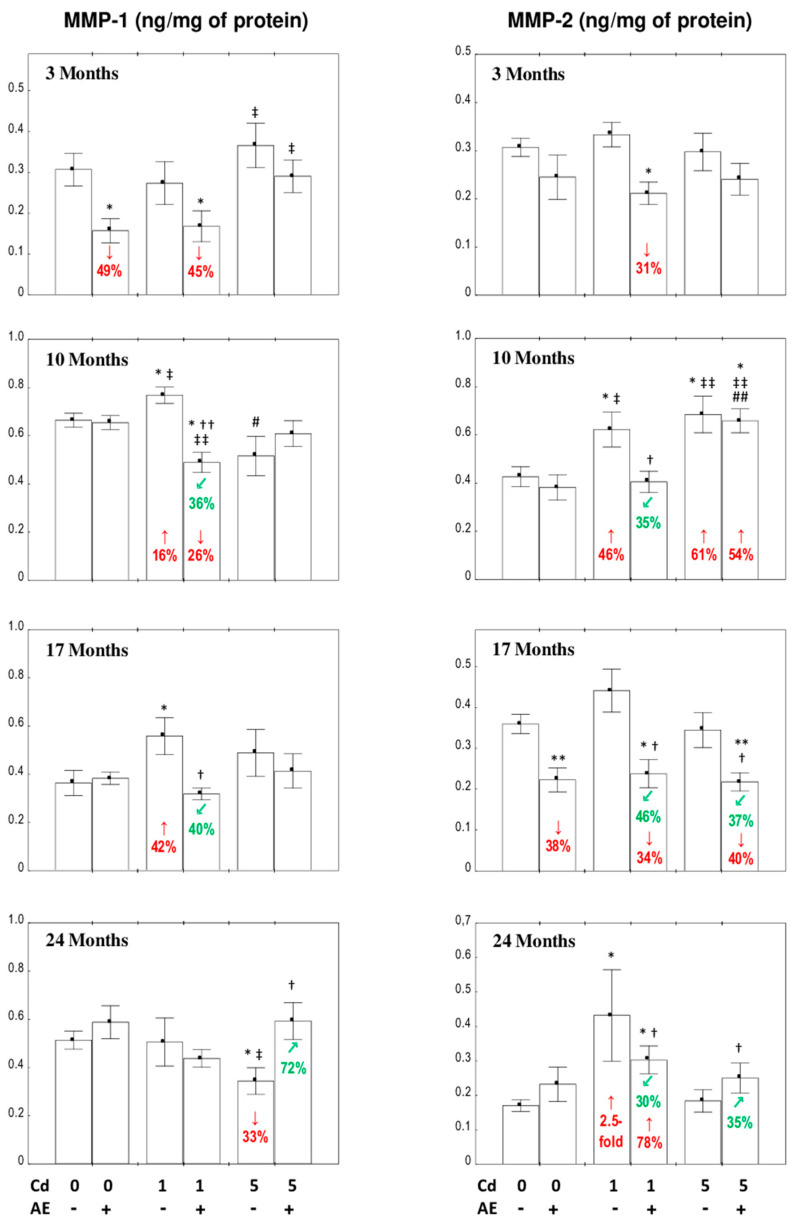
The impact of *Aronia melanocarpa* L. berry extract (AE) on the concentrations of matrix metalloproteinase-1 (MMP-1) and matrix metalloproteinase-2 (MMP-2) in the livers of rats exposed to cadmium (Cd). The animals were treated with Cd at the concentrations of 0, 1, and 5 mg Cd/kg in their diets and/or 0.1% aqueous AE (+) or not (−). Data are shown as mean ± SE for eight rats, except for seven animals in the AE, Cd_1_, and Cd_5_ groups after 24 months. Statistically significant differences (Kruskal–Wallis post hoc test): * p < 0.05 and ** *p* < 0.01 vs. control group; ^†^ *p* < 0.05 and ^††^ *p* < 0.01 vs. appropriate Cd group; ^‡^ *p* < 0.05 and ^‡‡^ *p* < 0.01 vs. AE group; ^#^ *p* < 0.05 and ^##^ *p* < 0.01 vs. appropriate group maintained on the 1 mg Cd/kg diet (Cd_1_ group or Cd_1_ + AE group). Numerical values in bars express the percentage changes or factors of changes in comparison to the control group (↑, increase; ↓, decrease) or the appropriate group exposed to Cd alone (↗, increase; ↙, decrease).

The consumption of AE alone during the 24-month experimental period had no impact on the liver concentration of MMPs, except for a drop in the concentration of MMP-1 after 3 and MMP-2 after 17 months (Figure 6).

The low-level dietary intoxication with Cd for 10 and 17 months increased the concentration of MMP-1 in the liver (by 16% and 42%, respectively), whereas the 24-month moderate intoxication with this xenobiotic led to its decrease (by 33%) (Figure 6). The supplementation with AE under the maintenance on the 1 mg Cd/kg diet for three months decreased the concentration of MMP-1 vs. the control group (Figure 6), and this was caused by the independent effect of the extract (*F* = 9.733, *p* < 0.01) (Table S3). Furthermore, the consumption of the extract for 10 months at this level of Cd treatment not only allowed the prevention of this toxic-element-caused increase in the concentration of MMP-1, but also decreased the level of this parameter compared to its proper value (by 26%); these resulted from its independent action (*F* = 18.31, *p* < 0.001) and interaction with Cd (*F* = 15.90, *p* < 0.001) (Table S3). The intake of AE during the 17- and 24-month treatment with Cd provided complete protection from this xenobiotic-caused alteration in the concentration of MMP-1 (Figure 6). The beneficial effect at the 17-month low-level treatment with Cd was caused by the independent impact of AE (*F* = 4.966, *p* < 0.05) and its interactive action with Cd (*F* = 6.198, *p* < 0.05), while after the moderate intoxication for 24 months, it was the main effect of the extract (*F* = 7.062, *p* < 0.05) (Table S3).

In the animals fed with the diet containing 1 mg Cd/kg, the concentration of MMP-2 was higher after 10 and 24 months (by 46% and 2.5-fold, respectively) and the AE totally or partially, respectively, protected from these changes (Figure 6). Moreover, the co-administration of chokeberry extract for 3 and 17 months decreased the concentration of MMP-2 in the rats exposed to the 1 mg Cd/kg diet compared to the control group (Figure 6). The only change in the concentration of MMP-2 with the moderate exposure to Cd was an increase (by 61%) in its value after 10 months. The AE co-administration for 10 months did not prevent this change, whereas its longer consumption decreased the concentration compared to both the control and Cd_5_ groups after 17 months and increased compared to the Cd_5_ group after 24 months (Figure 6). The modifying impact of the administration of AE to the animals exposed to Cd on the concentration of MMP-2 in the liver was the main effect of the extract’s action (*F* = 5.838–21.48, *p* < 0.05–0.001) (Table S3).

#### 3.5.2. The Concentrations of TIPM-1 and TIMP-2 in the Liver

**Figure 7:**
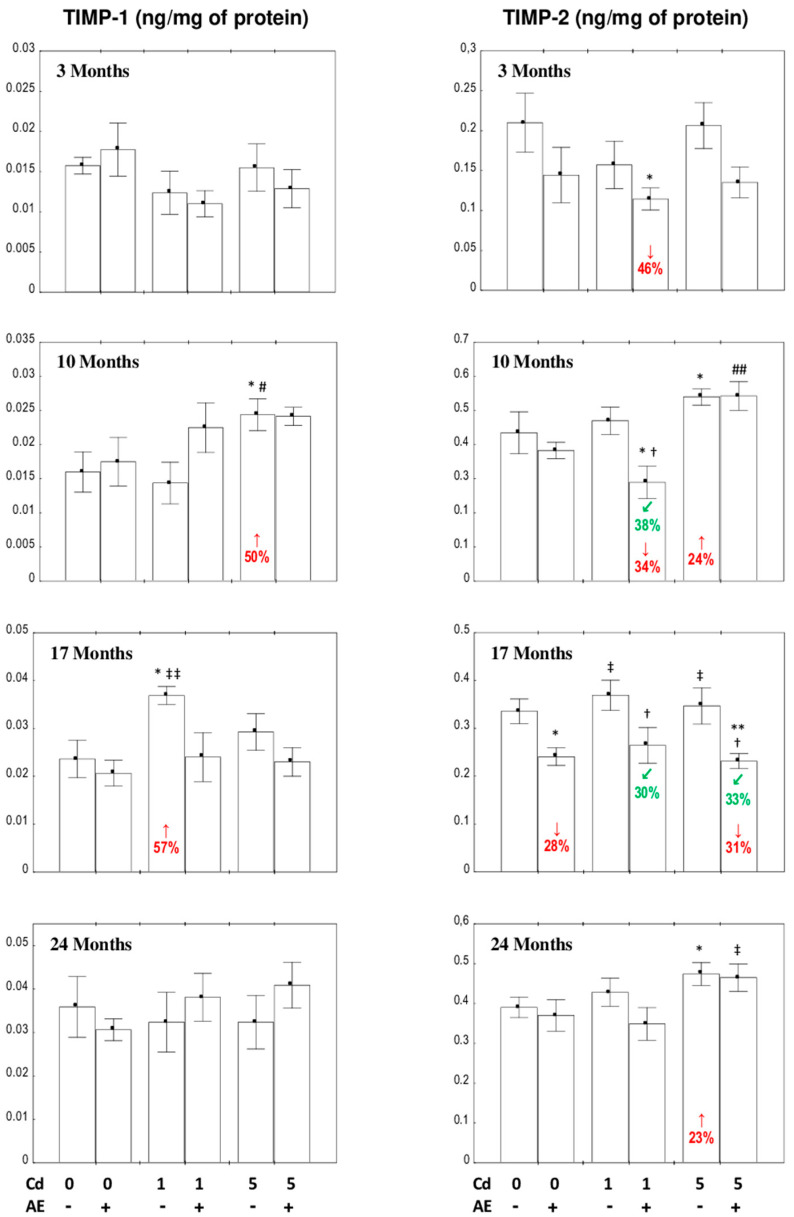
The impact of *Aronia melanocarpa* L. berry extract (AE) on the concentrations of tissue metalloproteinase inhibitor-1 (TIMP-1) and tissue metalloproteinase inhibitor-2 (TIMP-2) in the livers of rats exposed to cadmium (Cd). The animals were treated with Cd at the concentrations of 0, 1, and 5 mg Cd/kg in their diets and/or 0.1% aqueous AE (+) or not (−). Data are shown as mean ± SE for eight rats, except for seven animals in the AE, Cd_1_, and Cd_5_ groups after 24 months. Statistically significant differences (Kruskal–Wallis post hoc test): * p < 0.05 and ** *p* < 0.01 vs. control group; ^†^ *p* < 0.05 vs. appropriate Cd group; ^‡^ *p* < 0.05 and ^‡‡^ *p* < 0.01 vs. AE group; ^#^ *p* < 0.05 and ^##^ *p* < 0.01 vs. appropriate group maintained on the 1 mg Cd/kg diet (Cd_1_ group or Cd_1_ + AE group). Numerical values in bars express the percentage changes in comparison to the control group (↑, increase; ↓, decrease) or the appropriate group exposed to Cd alone (↙, decrease).

The only change in the concentrations of TIMP-1 and TIMP-2 in the livers of rats consuming AE alone was a decrease (by 28%) in TIMP-2 after 17 months of the study (Figure 7).

The concentration of TIMP-1 was unchanged at both levels of Cd treatment, except for its increase after 17 (by 57%) and 10 (by 50%) months of the exposure, respectively, whereas the use of AE provided total protection against the impact of this xenobiotic (Figure 7).

The low-level exposure to Cd did not affect the concentration of TIMP-2; however, the co-administration of AE decreased the value of this parameter vs. the control group after 3 and 10 months (by 46% and 34%, respectively) and vs. the Cd_1_ group after 10 and 17 months of the investigation (by 38% and 30%, respectively) (Figure 7). The 10 and 24-month maintenance on the diet containing 5 mg Cd/kg increased (by 24% and 23%, respectively) the concentration of TIMP-2. The simultaneous application of AE allowed the complete prevention of the increase in the concentration of this parameter after 10 and 24 months and decreased its value after 17 months vs. the control (by 31%) and Cd_5_ groups (by 33%) (Figure 7).

Generally, the liver concentration of TIMPs was independent of the intensity of exposure to Cd, except for the higher concentration of TIMP-1 (by 71%) in the Cd_5_ group vs. the Cd_1_ group and of TIMP-2 (by 88%) in the Cd_5_ + AE group vs. the Cd_1_ + AE group after 10 months of the experiment (Figure 7).

The ANOVA/MANOVA analysis revealed that the influence of AE on the concentrations of TIMP-1 and TIMP-2 resulted from the independent impacts of the extract components (*F* = 16.98, *p* < 0.001 and *F* = 6.593–16.53, *p* < 0.05–0.001, respectively) (Table S4). The two-way analysis of variance showed a lack of an independent impact of AE and/or its interactive action with Cd (Table S4) on TIMP-1 after 10 months of the moderate intoxication in spite of the visible impact of its administration recognized by the Kruskal–Wallis post hoc test (Figure 7).

#### 3.5.3. The Ratios of the Concentrations of MMP-1/TIMP-1, MMP-2/TIPM-2, and MMP-2/TIMP-1 in the Liver

**Figure 8:**
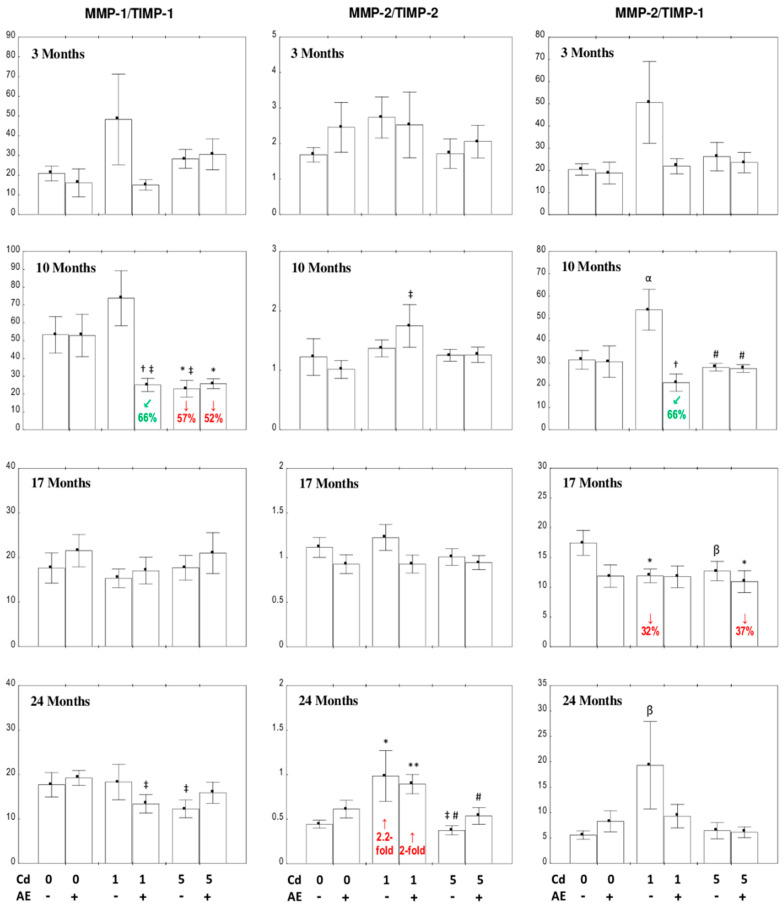
The impact of *Aronia melanocarpa* L. berry extract (AE) on the ratios of matrix metalloproteinase-1 (MMP-1)/tissue metalloproteinase inhibitor-1 (TIMP-1), matrix metalloproteinase-2 (MMP-2)/tissue metalloproteinase inhibitor-2 (TIMP-2), and MMP-2/TIMP-1 in the livers of rats exposed to cadmium (Cd). The animals were treated with Cd at the concentrations of 0, 1, and 5 mg Cd/kg in their diets and/or 0.1% aqueous AE (+) or not (−). Data are shown as mean ± SE for eight rats, except for seven animals in the AE, Cd_1_, and Cd_5_ groups after 24 months. Statistically significant differences (Kruskal–Wallis post hoc test): * p < 0.05, ** *p* < 0.01, ^α^ *p* < 0.06, and ^β^ *p* < 0.08 vs. control group; ^†^ *p* < 0.05 vs. appropriate Cd group; ^‡^ *p* < 0.05 vs. AE group; ^#^ *p* < 0.05 vs. appropriate group maintained on the 1 mg Cd/kg diet (Cd_1_ group or Cd_1_ + AE group). Numerical values in bars express the percentage changes or factors of changes in comparison to the control group (↑, increase; ↓, decrease) or the appropriate group exposed to Cd alone (↙, decrease).

The consumption of AE alone did not affect the ratios of MMP/TIMP concentrations in the liver during the whole experiment (Figure 8).

The only changes in the values of MMP-1/TIMP-1 ratios were a decrease in this parameter (by 66%) in the Cd_1_ + AE group vs. the Cd_1_ group and in the Cd_5_ and Cd_5_ + AE groups (by 57% and 52%, respectively) in comparison to the control group after 10 months (Figure 8). The MMP-2/TIMP-2 ratio was increased only in the Cd_1_ and Cd_1_ + AE groups (2.2- and 2-fold, respectively) after 24 months of Cd exposure (Figure 8). The ratio of MMP-2/TIMP-1 underwent reduction (by 66%) in the Cd_1_ + AE group in comparison to the Cd_1_ group after 10 months (Figure 8). The feeding with the 1 mg Cd/kg diet decreased (by 32%) the value of this ratio after 17 months, whereas the extract application offered complete protection from this effect of Cd (Figure 8). After 17 months of the study, the MMP-2/TIMP-1 ratio was also decreased (by 37%) in the Cd_5_ + AE group vs. the control group (Figure 8). Moreover, a clear tendency to increase in the value of this ratio occurred in the animals treated with the 1 mg Cd/kg diet for 10 and 24 months (*p* < 0.06 and *p* < 0.08, respectively); a drop due to the 17-month exposure to the 5 mg Cd/kg diet also occurred (*p* < 0.08) (Figure 8).

The influence of the extract co-administration on the ratio of MMP-1/TIMP-1 in the Cd_1_ + AE group resulted from the independent action of its ingredients (*F* = 4.834, *p* < 0.05) and their interactive action with Cd (*F* = 4.651, *p* < 0.05). The effect of simultaneous treatment with AE and Cd on the MMP-1/TIMP-1 ratio in the Cd_5_ + AE group, as well as on the MMP-2/TIMP-2 ratio in the Cd_1_ + AE group, was caused by this xenobiotic’s independent action (*F* = 11.91, *p* < 0.05 and *F* = 6.427, *p* < 0.05, respectively). The impact of chokeberry extract on the MMP-2/TIMP-1 ratio in the Cd_1_ + AE group after 10 months stemmed from the independent action of the extract (*F* = 6.706, *p* < 0.01), as well as its interaction with Cd (*F* = 6.107, *p* < 0.01). The ANOVA/MANOVA analysis showed no effect of AE or its interactive impact with this heavy metal on the MMP-2/TIMP-1 ratio after 17 months of the study.

In general, there were no differences between the MMPs/TIMPs ratios depending on the intensity of the treatment with Cd (Figure 8). However, lower values of the MMP-2/TIMP-2 ratio were noted in the Cd_5_ group vs. the Cd_1_ group (by 62%) and in the Cd_5_ + AE group compared to the Cd_1_ + AE group (by 40%) after 24 months. Moreover, the MMP-2/TIMP-1 ratio in the Cd_5_ group was lower (by 48%) vs. the Cd_1_ group, whereas the value of this ratio after 10 months in the Cd_5_ + AE group was higher compared to the Cd_1_ + AE group (by 29%) (Figure 8).

### 3.6. The Concentrations of MMPs and TIMPs in the Serum

#### 3.6.1. The Concentrations of MMP-1 and MMP-2 in the Serum

The consumption of AE alone did not influence the concentrations of MMP-1 and MMP-2 in the serum (Table 4).

The low-level intoxication with Cd increased (by 10%) the concentration of MMP-1 after 17 months and decreased (by 4.8%) after 24 months (Table 4). The exposure to the 5 mg Cd/kg diet increased this parameter after both 17 and 24 months (by 12% and 18%, respectively; Table 4). The feeding with the 1 mg Cd/kg diet led to an increase in MMP-2 concentration after 24 months (by 40%), while the moderate exposure to this xenobiotic increased the concentration after 17 and 24 months of the research (by 97% and 45%, respectively) (Table 4). The administration of AE during Cd intoxication entirely counteracted the impact of this xenobiotic on the concentration of both MMPs (Table 4).

The only differences in the serum concentrations of MMP-1 and MMP-2 between the groups of rats that received Cd alone or were co-administered with AE were higher values of MMP-1 (by 24%) and MMP-2 (by 52%) in the Cd_5_ group than in the Cd_1_ group after 24 months and 17 months, respectively, as well as lower (by 18%) concentration of MMP-2 in the Cd_5_ + AE group than in the Cd_1_ + AE group after 24 months of the study (Table 4).

The AE’s influence on the serum levels of MMP-1 and MMP-2 in the animals intoxicated with Cd was caused by the independent impact of its ingredients (*F* = 4.981–9.626, *p* < 0.05–0.01) and/or their interaction with this toxic metal (*F* = 8.688–34.42, *p* < 0.01–0.001) (Table S5), which had an antagonistic character. The two-way analysis of variance revealed the lack of an independent impact of AE and the interaction of its ingredients with Cd (Table S5) on the concentration of MMP-1 in the serum in the Cd_1_ + AE group after 24 months, contrary to its beneficial effect disclosed by the Kruskal–Wallis analysis (Table 4).

#### 3.6.2. The Concentrations of TIMP-1 and TIMP-2 in the Serum

The concentrations of TIMP-1 and TIMP-2 in the serum were unchanged due to the treatment with AE alone for up to 24 months (Table 5).

The dietary intoxication with Cd at the concentrations of 1 and 5 mg Cd/kg decreased the concentration of TIMP-1 after 17 and 24 months (by 44–49%), whereas the concentration of TIMP-2 was decreased after 10 months (by 15%) of the low-level treatment, as well as after 10 and 24 months (by 15% and 24%, respectively) of moderate intoxication with this xenobiotic (Table 5). The supplementation with AE completely protected against these effects of Cd, except for partial protection in the TIMP-1 concentration after 17 months of simultaneous administration of the 5 mg Cd/kg diet and the extract (Table 5).

No differences in the serum concentrations of TIMP-1 and TIMP-2 were noted between the appropriate groups exposed to Cd alone or co-administered with this xenobiotic and AE, except for a lower (by 20%) level of TIMP-2 in the Cd_5_ group than in the Cd_1_ group and a higher (by 7%) concentration of this parameter in the Cd_5_ + AE group than in the Cd_1_ + AE group after 24 months (Table 5).

The ANOVA/MANOVA analysis showed that the impact of AE on the concentrations of TIMP-1 and TIMP-2 in the serum of the rats was caused by the interaction of its components with Cd (*F* = 8.785–42.86, *p* < 0.01–0.001) (Table S6), apart from the Cd_5_ + AE group after 24 months, where the favorable influence of the extract on TIMP-2 concentration was caused by both independent action of its ingredients (*F* = 36.55, *p* < 0.001) and their antagonistic interaction with this toxic metal (*F* = 24.97, *p* < 0.001) (Table S6).

### 3.7. Mutual Dependencies between Collagen, MMPs, and TIMPs in the Liver and Serum

Numerous positive or negative mutual relationships between the parameters of collagen metabolism in the liver were noted (Table 6).

The dependencies between the concentrations of MMP-1, MMP-2, TIMP-1, and TIMP-2 in the liver and serum included only a negative correlation between the liver concentration of MMP-2 and the serum concentration of MMP-2 (*r* = −0.322, *p* < 0.001).

### 3.8. Mutual Dependencies between Indices of Collagen Metabolism in the Liver and Cd Concentration in This Organ

Positive correlations were disclosed between the liver Cd concentration and the concentrations of TIMP-1 and TIMP-2 (*r* = 0.172, *p* < 0.05 and *r* = 0.198, *p* < 0.01, respectively), the expression of collagen III at the protein level (*r* = 0.280, *p* < 0.001), and the ratio of collagen III and collagen I at the protein level in this organ (*r* = 0.288, *p* < 0.001). A negative correlation was noted between the liver concentration of this metal and the expression of collagen I at the protein level (*r* = −0.170, *p* < 0.05). There was no correlation between the concentration of Cd in this organ and total collagen concentration, collagen III and I expression and their ratio at the mRNA level, or MMP-1 and MMP-2 concentrations.

## 4. Discussion

The present investigation provides the first evidence not only for the negative impact of low chronic intoxication with Cd on collagen metabolism in the liver, but also for the beneficial influence of a polyphenol-abundant chokeberry extract against this xenobiotic-mediated destruction in the metabolism of this basic component of the hepatic ECM. Disturbances in collagen metabolism in the liver, especially collagens I and III, regardless of the cause, may have very serious consequences [22,23,24,29]. The liver, which plays a key role in the body’s homeostasis, is responsible for the synthesis, metabolism, storage, and redistribution of nutrients [34]. Damage to this organ may be induced by chronic infection with hepatotoxic viruses (mainly hepatitis B and C viruses) and autoimmune damage, as well as metabolic causes and xenobiotics; nonetheless, it has a very high regenerative capacity [35]. However, if the injury of the liver persists, such as in liver fibrosis, an excessive accumulation of collagenous ECM often occurs, which can also be reversible in the early stages, before normal liver architecture is disturbed and its function is eventually impaired [22,23,28].

Cd is a well-recognized hepatotoxic agent [1,9,10,11,12,13,18,19,20,21,28,30,36]. The mechanism of its damaging action on the liver is complex [1,9,12,13,18,19,20,21,28,30,36]; however, it is still not fully explained, especially at low intoxication. Until now, little has been known about this xenobiotic’s action on the ECM components in the hepatic tissue, and about the regulation routes responsible for the metabolism of ECM, such as MMPs and TIMPs [18,19,20,21,28,36]. The findings of the current investigation indicate that the unfavorable influence of low and moderate chronic intoxication with Cd on the liver relies on destroying collagen metabolism, and that this impact is related to dysregulation of the MMP/TIMP system. MMPs and TIMPs have an important role in maintaining the balance in collagen metabolism, disturbance of which may be potentially harmful [22,23,29]. It is worth emphasizing that, at both levels of exposure, this xenobiotic significantly altered the expression of collagen I and III at the levels of mRNA and protein and the ratio of collagen III to collagen I at both levels, and also dysregulated the MMP/TIMP system in the liver. However, despite this, it had only a slight and temporary effect, if any, on the concentration of total collagen in this organ. Taking into account the fact that, in the rats maintained on the diet containing 5 mg Cd/kg for three months, the expression of collagen I at the mRNA and protein levels was decreased, while that of collagen III at these levels was increased or unaffected, respectively, and the concentrations of enzymes degrading collagen—MMP-1 and MMP-2—did not change, it seems that the increase in the concentration of total collagen noted in these rodents could be caused by enhanced expression of other types of collagen present in this organ, such as collagens IV, V, and VI [22,29], rather than a lower level of this protein degradation, as in the case of fibrosis, resulting from an increase in collagen deposition [23]. Although collagens I and III are among the main types of collagen that, according to some authors, constitute 40% each of total collagen in the liver [37,38]—or, according to Masuda et al. [39], collagen I predominates—other collagens may also affect the concentration of total collagen. It has been shown that the expression of collagen types IV and V, forming up to 10% of the total collagen in the liver, may increase even more than the expression of collagen I or III [40,41].

The clear tendency to increase in the concentration of total collagen noted after the treatment with the 1 mg Cd/kg diet for 17 months might be a result of the enhanced expression of collagens I and III at the levels of mRNA and protein. The lack of the impact of Cd on the concentration of total collagen at almost all time points with the low-level and moderate treatments with this toxic element and the simultaneous clearly evident influence on the expression of collagens I and III at the mRNA and protein levels may be explained by the different directions of the impact of this xenobiotic on the expression of collagens I and III, as well as its possible influence on other types of collagen present in this organ [22,29]. The absence of significant changes in the concentration of total collagen due to the 3- and 10-month intoxication with Cd, except for its increase after three months of the moderate treatment, can be a result of the decreased expression of collagen I and increased expression of collagen III. At the present stage of our research, we cannot yet explain why the concentration of total collagen after 17 and 24 months remained unaltered, except for the increasing tendency in the group fed with the diet containing 1 mg Cd/kg for 17 months, in spite of markedly influenced collagen I and collagen III expression at the level of protein. The opposite effect of Cd on collagens I and III after 17 and 24 months and the absence of significant changes in the total concentration of this protein can be explained by changes in the expression of other collagen types or by the varying degradation of these collagens in different periods of the treatment with Cd. Similar changes in the most abundant collagen types I and III, as well as collagen types IV, V, and VI, have also been observed at various stages of liver fibrosis. All these collagens are known to be altered in quantity and quality in fibrosis of this organ [23].

Except for the 24-month period of the experiment, where discrepancies in changes in collagen I expression at the levels of mRNA and protein under the influence of Cd were detected, in all other periods of rat exposure to Cd, changes in the expression of mRNA were consistent with those of the protein expression of both types of collagens. The decrease in the amount of collagen I after 24 months, despite the significant increase in collagen mRNA at the transcription stage under the influence of both levels of intoxication with Cd, suggests that the effect of this element on post-transcriptional regulation of collagen biosynthesis is dependent on, among other things, the pool of free proline and prolyl-tRNA concentration, as well as the expression and activity of many enzymes important in the folding and modification of the collagen triple helix, processing, and secretion of this protein into the ECM [40,42].

It is difficult to explain why, under the influence of Cd during the 3- and 10-month exposure, the drop in collagen I expression and the enhancement in collagen III expression were noted. It is possible that these are due to the diverse mechanisms of transcriptional and/or post-transcriptional regulation of both types of collagen, which may be affected by Cd. In addition, various signaling pathways may take part in the processes of regulation of the transcription and translation of both types of collagen in the liver cells [43]. Stimulation of biosynthesis of collagen I at the mRNA and protein levels is regulated by signal transduction involving protein kinase A, Src non-receptor tyrosine kinases, and extracellular signal-regulated kinase (ERK1/2), while stimulation of mRNA and protein of collagen III is dependent on the activation of p38 mitogen-activated protein kinase (MAPK). It is also possible that under the exposure to Cd, collagen III may undergo slower degradation than that of type I, despite the increased concentration of MMP-2 [44].

Since the proper ratio of collagens I and III is critical for forming the viscoelastic heterotypic collagen fibers contributing to the appropriate mechanical properties of the ECM and determining cell physiology and tissue homeostasis [22,45], it was revealed in the present study that the feeding with the diets containing 1 and 5 mg Cd/kg not only influenced the expression of both types of collagen, but also changed the ratio of collagen III to collagen I, which provides clear evidence for the unfavorable impact of the low intoxication with this heavy metal on the ECM in the liver. Due to differences in their elasticity and dynamic properties, collagen III is able to soften fibrils of collagen I, but it depends on the relative concentration of collagen type III with respect to collagen I monomers [26,27]. The changes in the content of these collagens and their ratio, detected in our research under the influence of Cd, which probably resulted in changes in fibril geometry, may influence the biomechanical properties of collagen fibrils and consequently affect the function of this organ. The significant alterations in the ratios of both major types of collagen in the liver revealed during the 3- and 10-month treatment with Cd may contribute to the changes in the structure and biomechanical properties of heterotypic fibrils of collagen. Similarly, the longer intoxication with Cd (17 and 24 months), which did not cause such significant changes in the ratio of both types of collagen, but also led to important quantitative alterations (excess after 17 months and deficiency after 24 months of both major collagens), might certainly destroy the composition and properties of the liver ECM and consequently disturb the hepatic tissue homeostasis. In our study, the livers in young control rats (after 3 and 10 months) had less collagen III in relation to collagen I, and with aging, the ratio of both collagens was equal to about 1, and these values are in agreement with the data in the literature [37,38,39]. The enhanced proportion of types III and I procollagen mRNA has been revealed in the course of dimethylnitrosamine-induced liver damage; however, as in the current investigation, no increase was noted in the content of total collagen in the liver [37].

An important result of this investigation is the demonstration of the disturbances in the metabolism of collagen in the liver at low Cd concentration in this organ and in the blood, amounting to 0.1447 ± 0.0093 μg/g and 0.1884 ± 0.0100 μg/L, respectively [17]. This finding may be very concerning because such concentrations of this xenobiotic are noted in the inhabitants of economically developed areas [8,9,10,11]. Moreover, the mutual dependencies disclosed between the liver concentration of Cd and the estimated indices of collagen metabolism show that destroying the metabolism of this main component of the ECM in this organ will intensify with increasing accumulation of this xenobiotic in the body. Another significant outcome of the present research is showing that the Cd-caused disorders in the metabolism of collagens I and III in the hepatic tissue appeared already as a result of the shortest and lowest of the investigated treatments with this toxic metal. These indicate that Cd may negatively influence the ECM of the hepatic tissue already at the early stage of exposure. It is also worth noting that generally, the effect of this heavy metal on the investigated parameters of collagen metabolism did not depend on the intensity of exposure, but in the cases where the difference was noted, the effect was usually more significant at the moderate than at the low-level intoxication.

The possible mechanism of Cd action on collagen metabolism in the liver includes a direct and indirect impact of this heavy metal. In addition, as revealed in the present investigation, the ability of Cd to influence the synthesis of this protein and the MMP/TIMP system controlling collagen degradation, the data in the literature indicate that this heavy metal may also act on collagen metabolism by inducing HSC, profibrogenic cytokines, and production of ROS [20,28,46]. Recently, we revealed that maintaining diets containing 1 and 5 mg Cd/kg disturbed the oxidative/reductive status in the liver and resulted in development of oxidative stress and oxidative modifications of cellular macromolecules such as proteins, lipids, and DNA in this organ [12,13]. Thus, it seems possible that the effect of this xenobiotic noted in the present study on the status of collagen may also be related to its prooxidative properties.

The impact of Cd on MMPs and TIMPs in the liver varied in different time points and depended on the level of the exposure; however, if this effect occurred, it (except for MMP-1 in the Cd_5_ group after the 24-month treatment) consisted in an elevation in the values of the investigated indices of the MMP/TIMP system almost every time. Although the ratios of MMP-1/TIMP-1, MMP-2/TIMP-2, and MMP-2/TIMP-1 (this ratio was calculated since TIMP-1 influences not only the activity of MMP-1, but also that of MMP-2 [47]) were only changed temporarily, or tended to be changed, due to the treatment with Cd, the impacts on the concentrations of particular MMPs and their inhibitors show that the MMP/TIMP system was affected by this xenobiotic. The lack of significant changes in the above-mentioned ratios of MMPs and TIMPs may suggest that, generally, in spite of the modified concentrations of particular MMPs and TIMPs in the hepatic tissue, the MMP/TIMP balance might be maintained, and thus, the concentration of total collagen in this organ also remained almost unchanged. According to our knowledge, in the available literature, there are no data on the impact of low and moderate chronic intoxication with this toxic element on MMPs and TIMPs in the liver. Only deMoura et al. [36] have revealed that an eight-week intoxication with this xenobiotic had no impact on the activity of MMP-2 in rat livers. The findings of this study are in accordance with the findings of other authors showing that Cd exposure may induce MMPs and TIMPs in various tissues and organs in the body, as well as in cell cultures [31,48,49]. On the contrary, there are also data showing that Cd may decrease the activity of MMP-2 in the prostate and testis and increase the activity of this metalloproteinase in the brain [48,50]. The lack of correlations between the liver and serum levels of MMP-1, TIMP-1, and TIMP-2 show that a measurement of the serum concentrations of these variables—unlike MMP-2, in the case of which the negative correlation between its concentration in the liver and serum occurred—cannot have predictive value regarding the MMP/TIMP status in the liver. These may be explained by the fact that the serum concentration of these parameters is the reflection of Cd action in the whole organism.

The practically useful and, thus, the most significant result of the present work is the disclosure that AE may be effective in preventing Cd-induced alterations in the expression of collagens I and III at the levels of mRNA and protein in the liver, as well as in the concentrations of MMPs and their tissue inhibitors in the liver and serum. What is more, an important outcome of the study is showing that the daily consumption of 31.1–154.7 mg AE/kg b.w. alone for up to 24 months generally (apart from the temporary alteration of some parameters) does not change the investigated parameters, which is a significant advantage of the extract when considering its influence on the homeostasis of the ECM in physiological conditions. The findings of the two-way analysis of variance enable us to conclude that the favorable effects of the chokeberry extract on the liver metabolism of collagens I and III as well as the concentrations of MMPs and TIMPs in this organ and in the serum of the animals maintained on diets containing 1 and 5 mg Cd/kg were caused by the independent impact of the extract’s ingredients and their interaction, which was antagonistic in character, with this xenobiotic. The independent action of AE may result from its high antioxidative potential and the ability to combat oxidative stress [3,12,13]; as a result, it prevents oxidative-mediated alteration in collagen expression and the concentrations of MMPs and TIMPs. The interactive action of the extract and Cd was mediated by the chelating properties of its ingredients [3,4,17]. Chelation of Cd^2+^ by, first of all, polyphenols, as well as fiber (including pectins), decreases the absorption from the digestive tract and retention in the body of this element as well as, thereby, its accumulation in the liver [3,4,17]. Indeed, Cd concentration in this organ in the rats supplemented with AE during the treatment with this toxic metal was lower by 10–37% compared to those that only received Cd (Table S7). The antagonistic interactive impact of Cd and chokeberry extract ingredients on the investigated parameters may stem from the extract-mediated reduction of the concentration of this toxic element in the liver. Numerous correlations disclosed between the estimated indices of collagen metabolism and Cd concentration in the liver show that the adverse effect of this heavy metal on the status of collagen in the liver will ameliorate with decreasing Cd accumulation in this organ.

Although the impact of AE on collagen and the MMP/TIMP system in the liver under exposure to Cd has not been investigated until now, polyphenolic compounds and polyphenol-rich extracts have been shown to improve collagen homeostasis in the organism, including the hepatic tissue [15,18,21,31,32,51,52,53,54,55,56,57,58,59,60]. In our previous investigation, we revealed that AE prevented Cd-induced increase in the serum concentration of carboxy-terminal crosslinking telopeptides of type I collagen as a product of collagen degradation and a biomarker of bone turnover [15]. Anthocyanins from *A. melanocarpa* were shown to exert protective action regarding carbon tetrachloride (CCl_4_)-mediated fibrosis of the liver in mice, and the mechanism of this effect included, first of all, inhibition of the overexpression of transforming growth factor-β1 (TGF-β1) (which can activate HSC), an influence on the TGF-β/Smad signal transduction pathway, which is responsible for the profibrotic effect of TGF-β1, and reduction in the expression of α-smooth muscle actin (α-SMA; a marker of HSC activation) and collagen I, as well as a decrease in the expression of inflammatory cytokines (tumor necrosis factor-α—TNF-α and interleukin-1) [51]. Anthocyanins isolated from blueberries also provided protection from CCl_4_-induced liver fibrosis, which was an effect of reduction of ROS formation, suppression of the HSC activity, and downregulation of the expression of proinflammatory cytokines, as well as TIMP-1, collagen III, and α-SMA, and upregulation of matrix metalloproteinase-9 (MMP-9) expression [52], while these compounds—occurring in purple-fleshed sweet potato—caused inhibition of the expression of collagens I and III, as well as TGF-β1, α-SMA, and TNF-α in the livers of rats exposed to dimethylnitrosamine [53]. Cyanidin-3-O-β-glucoside and chlorogenic acid, which are the main polyphenols present in AE [2,15], were reported to exert an anti-fibrotic effect in the liver [54,55,56]. Jiang et al. [54] revealed that cyanidin-3-O-β-glucoside (extracted from the skin of black rice—*Oryza sativa* L. indica) had a protective effect on the CCl_4_-induced liver fibrosis in mice and on the activation of HSC isolated from mice by downregulation of the collagen type I gene, as well as an increase in the expression of MMP-2. Shi et al. [55] noted that chlorogenic acid markedly alleviated the content of hydroxyproline and the expression of α-SMA, collagen I, collagen III, and TIMP-1, attenuating the CCl_4_-caused fibrosis of the liver in rats, and suggested that this effect is, at least in part, connected with the suppression of oxidative stress. Similarly, Yang et al. [56] disclosed that chlorogenic acid reduced the expression of α-SMA and collagen I in the liver tissue and TGF-β1 in the serum, and lessened the degree of the liver fibrosis induced by CCl_4_ in rats. Moreover, quercetin, a flavonoid occurring in chokeberries [2], was demonstrated to prevent against an enhancement in the amount of collagen in the hepatic tissue of rats intoxicated with Cd [18]. The chokeberry extract is rich in phytochemicals of the polyphenol family that possess an inhibitory action on the MMPs, such as naringenin, quercetin, kaempferol, proanthocyanidin, caffeic acid, chlorogenic acid, and gallic acid [2,4,15,57,58,59,60]. A similar effect is expressed by non-polyphenol components of AE. In studies in patients with diabetic nephropathy, the administration of vitamin E caused a reduction in the serum concentration of MMP-2, MMP-9, and TNF-α [61], while zinc was shown to decrease the Cd-induced expression of MMP-1 in synoviocytes [62]. De Lima et al. [21] noted that a polyphenol-rich extract from *Mimosa caesalpiniifolia* caused a qualitative reduction of the liver fibrosis in rats treated with Cd by inhibiting the extracellular matrix synthesis. Grape seed (*Vitis vinifera*) [32] was shown to suppress this metal-induced deposition of collagen and decrease the expression of profibrogenic cytokines, including TGF-β1, in the prostate [32]. The available data on the protective impact of polyphenols and various products rich in these compounds against Cd-induced liver injury have been presented and discussed by us elsewhere [1].

The findings of the current investigation, together with the results of our recent research [12,13,17], provide evidence that AE counteracts Cd hepatotoxicity at multiple levels. Previously, we revealed that the favorable effect is connected with the chokeberry-extract-mediated decrease in the content of this metal in the liver and the prevention against oxidative stress development and its consequences, such as oxidative damage to the cellular macromolecules, as well as pathological changes in this organ’s morphological structure [12,13]. The extract’s administration under the exposure to the 1 and 5 mg Cd/kg diets makes all of the pathological changes in the morphology of the hepatic tissue less intense, or even completely prevented them (Table S1) [12]. The findings of the present study show that the protective action of AE on the liver also includes the protection from the destruction of the metabolism of collagen caused by Cd.

The facts that the liver is one of the main places for Cd accumulation in the body [17] and that damage to this organ is one of the main global problems of public health [63] create a need to look for efficient means to protect against Cd hepatotoxicity, and aronia products seems to be a promising solution to this issue. Moreover, the present investigation, together with our previous reports on the hepatoprotective ability of the extract from chokeberries with respect to intoxication with Cd [12,13], is one of the comprehensive studies conducted by our research team on the possibility of use of chokeberries to prevent damage to the target organs and systems due to lifelong exposure to Cd, reflecting the lifelong environmental exposure of the general population in industrialized countries, or to at least weaken these outcomes [12,13,14,15,16,17]. The results of these studies allow us to consider the chokeberry as a promising candidate to provide holistic protection of the organism against Cd toxicity.

Regardless of all the important and new findings, there are also some limitations of the present research. First of all, the study was performed on females due to their higher susceptibility to this toxic element than males [17]; therefore, the outcomes of the research refer to the female liver. The second issue is that there was no possibility to evaluate the time-related effects in the same animals, but rather in various subgroups (7–8 rodents each) of the same study groups. This may be the explanation for why some effects of the impact of Cd and/or chokeberry extract occurred or did not occur at some time points or only a tendency to change was observed. The main limitation of the present investigation is that the expression of liver collagens other than collagens I and III was not estimated, as well as the fact that we are unable to more completely explain the most possible mechanisms of the unfavorable influence of Cd and the protective role of AE on the metabolism of collagen in the liver. However, the aim of the current paper was not the investigation of these mechanisms, but, first of all, showing whether the repeated low and moderate intoxication with this xenobiotic affects collagen metabolism in the liver and whether the administration of AE may provide protection in this respect. The possible mechanisms of the impacts of both Cd and AE on the ECM of the liver will be evaluated by us in further studies.

In summary, the findings of the current investigation show, for the first time, that an extract from the berries of *A. melanocarpa*, which is a rich source of polyphenolic compounds, offers protection against disturbances in the expression of collagen I and collagen III at the levels of mRNA and protein in the liver, as well as deregulation of the MMP/TIMP system in the liver under low and moderate long-term intoxication with Cd. A very important outcome of the present study is also the finding that Cd may affect the synthesis of collagen and affect the MMP/TIMP system regulating this protein degradation at the treatment (1 mg Cd/kg diet), which reflects human environmental exposure in industrialized countries. The results provide another argument supporting the need to search for effective ways to protect against this toxic-metal-induced injury to various organs, including the liver. Moreover, following our previous investigations [12,13,14,15,16,17], the present findings provide further support of the effectiveness of the chokeberry extract in combating the unfavorable impact of Cd action in the organism, including in the hepatic tissue. We are aware that the possibility of eventual use of the berries of *A. melanocarpa* in the protection of the liver in humans exposed to Cd needs further investigation. Nevertheless, chokeberry products seem to be very promising in protection from the unfavorable effects of action of this xenobiotic.

## Figures and Tables

**Table 1:** Evaluation of the main and interactive effects of cadmium (Cd) and *Aronia melanocarpa* L. berry extract (AE) on the expression of collagen type I and collagen type III at the messenger ribonucleic acid (mRNA) level in the livers of rats ^1,2^.

Duration (Months)	1 mg Cd/kg Diet + AE				5 mg Cd/kg Diet + AE			
	Main Effect of Cd	Main Effect of AE	Interactive Effect of Cd + AE	Cd + AE Effect vs. Cd Effect + AE Effect *Possible Character of* *Cd–AE Interaction*	Main Effect of Cd	Main Effect of AE	Interactive Effect of Cd + AE	Cd + AE Effect vs. Cd Effect + AE Effect *Possible Character of* *Cd–AE Interaction*
**Collagen I mRNA**								
**3**	9.219 **	NS	22.12 ***	0 vs. −59 ^3^ + (−37) 0 vs. −96 *Antagonistic action*	21.07 ***	NS	21.44 ***	−37 vs. −64 + (−37) −37 vs. −101 *Antagonistic action*
**10**	7.277 *	NS	14.76 **	0 vs. −51 + 0 0 vs. −51 *Antagonistic action*	20.82 ***	NS	8.056 *	−34 vs. −67 + 0 −34 vs. −67 *Antagonistic action*
**17**	26.75 ***	8.803 **	42.48 ***	0 vs. 2-fold + (+1.33-fold) 0 vs. +3.33-fold *Antagonistic action*	14.57 **	NS	9.321 **	+43 vs. +85 + (+33) +43 vs. +118 *Antagonistic action*
**24**	23.86 ***	12.69 **	19.75 ***	0 vs. +4-fold + 0 0 vs. +4-fold *Antagonistic action*	23.76 ***	5.898 *	11.92 **	0 vs. +3.3-fold + 0 0 vs. +3.3-fold *Antagonistic action*
**Collagen III mRNA**								
**3**	25.52 ***	18.81 ***	NS	No interaction	13.81 **	NS	NS	No interaction
**10**	24.68 ***	4.851 *	11.26 **	+1.43-fold vs. +2.3-fold + 0 +1.43-fold vs. +2.3-fold *Antagonistic action*	122.5 ***	NS	NS	No interaction
**17**	8.179 **	NS	17.69 ***	0 vs. +80 + 0 0 vs. +80 *Antagonistic action*	44.79 ***	NS	15.19 ***	+1.64-fold vs. +2.3-fold + 0 +1.64-fold vs. +2.3-fold *Antagonistic action*
**24**	8.021 *	10.07 **	NS	No interaction	39.39 ***	6.196 *	NS	No interaction

^1^ The findings of the two-way analysis of variance (ANOVA/MANOVA) are expressed as *F* values and the level of statistical significance (*p*). *F* values with *p* < 0.05 are considered statistically significant (* *p* < 0.05, ** *p* < 0.01, *** *p* < 0.001). NS—not statistically significant (*p* > 0.05). ^2^ In order to evaluate the possible character of the interaction between Cd and AE, the effect disclosed at their co-administration was compared to the sum of the effects noted in separate treatments with these agents (Cd + AE effect vs. Cd effect + AE effect). The Cd effect, AE effect, and Cd + AE effect are shown as percentage changes or factors of changes (+, increase; −, decrease) of a measured parameter vs. the control group. Cd−AE interaction was recognized as antagonistic when the result of simultaneous treatment with Cd and AE was less than the mathematic sum of the results noted at their separate application. ^3^ The values represent percentage changes.

**Table 2:** Evaluation of the main and interactive effects of cadmium (Cd) and *Aronia melanocarpa* L. berry extract (AE) on the expression of collagen type I and collagen type III at the protein level in the livers of rats ^1,2^.

Duration (Months)	1 mg Cd/kg Diet + AE				5 mg Cd/kg Diet + AE			
	Main Effect of Cd	Main Effect of AE	Interactive Effect of Cd + AE	Cd + AE Effect vs. Cd Effect + AE Effect *Possible Character of Cd–AE Interaction*	Main Effect of Cd	Main Effect of AE	Interactive Effect of Cd + AE	Cd + AE Effect vs. Cd Effect + AE Effect *Possible Character of Cd–AE Interaction*
**Collagen I**								
**3**	NS	NS	13.88 **	0 vs. −19 ^3^ + 0 0 vs. −19 *Antagonistic action*	14.47 **	9.928 **	25.37 ***	0 vs. −31 + 0 0 vs. −31 *Antagonistic action*
**10**	232.4 ***	72.18 ***	50.73 ***	−10 vs. −34 + 0 −10 vs. −34 *Antagonistic action*	138.8 ***	34.42 ***	25.38 ***	−15 vs. −43 + 0 −15 vs. −43 *Antagonistic action*
**17**	19.91 ***	4.297 *	31.09 ***	+12 vs. +50 + (+17) +12 vs. +67 *Antagonistic action*	12.57 **	NS	17.61 ***	+14 vs. +33 + (+17) +14 vs. +50 *Antagonistic action*
**24**	9.685 **	7.746 *	9.121 **	0 vs. −27 + 0 0 vs. −27 *Antagonistic action*	14.23 **	16.55 ***	19.22 ***	0 vs. −32 + 0 0 vs. −32 *Antagonistic action*
**Collagen III**								
**3**	18.05 ***	5.407 *	27.16 ***	+33 vs. +51 + (+37) +33 vs. +88 *Antagonistic action*	10.82 **	39.33 ***	NS	No interaction
**10**	14.09 **	NS	38.58 ***	0 vs. +64 + (+33) 0 vs. +97 *Antagonistic action*	98.93 ***	NS	6.636 *	2-fold vs. +2.2-fold + (+1.33-fold) 2-fold vs. 3.53-fold *Antagonistic action*
**17**	148.9 ***	43.15 ***	217.8 ***	+17 vs. +80 + (+24) +17 vs. +104 *Antagonistic action*	150.3 ***	20.02 ***	121.2 ***	+29 vs. +86 + (+24) +29 vs. +110 *Antagonistic action*
**24**	14.62 **	8.997 **	NS	No interaction	54.56 ***	9.717 **	NS	No interaction

^1^ The findings of the two-way analysis of variance (ANOVA/MANOVA) are expressed as *F* values and the level of statistical significance (*p*). *F* values with *p* < 0.05 are considered statistically significant (* *p* < 0.05, ** *p* < 0.01, *** *p* < 0.001). NS—not statistically significant (*p* > 0.05). ^2^ In order to evaluate the possible character of the interaction between Cd and AE, the effect disclosed at their co-administration was compared to the sum of the effects noted in separate treatments with these agents (Cd + AE effect vs. Cd effect + AE effect). The Cd effect, AE effect, and Cd + AE effect are shown as percentage changes or factors of changes (+, increase; −, decrease) of a measured parameter vs. the control group. Cd–AE interaction was recognized as antagonistic when the result of simultaneous treatment with Cd and AE was less than the mathematic sum of the results noted in their separate applications. ^3^ The values represent percentage changes.

**Table 3:** Evaluation of the main and interactive effects of cadmium (Cd) and *Aronia melanocarpa* L. berry extract (AE) on the ratio of the expression of collagen type III to that of collagen type I at the mRNA and protein levels in the livers of rats ^1,2^.

Duration (Months)	1 mg Cd/kg Diet + AE				5 mg Cd/kg Diet + AE			
	Main Effect of Cd	Main Effect of AE	Interactive Effect of Cd + AE	Cd + AE Effect vs. Cd Effect + AE Effect *Possible Character of Cd–AE Interaction*	Main Effect of Cd	Main Effect of AE	Interactive Effect of Cd + AE	Cd + AE Effect vs. Cd Effect + AE Effect *Possible Character of Cd–AE Interaction*
**Collagen III/Collagen I Expression at the mRNA Level**								
**3**	14.45 **	7.268 *	13.65 **	0 vs. 3.8-fold + 0 0 vs. 3.8-fold *Antagonistic action*	30.75 ***	6.776 *	14.81 **	+1.89-fold vs. +3.8-fold + 0 +1.89-fold vs. +3.8-fold *Antagonistic action*
**10**	26.67 ***	13.19 **	24.01 ***	+1.55-fold vs. +4.6-fold + 0 +1.55-fold vs. +4.6-fold *Antagonistic action*	40.22 ***	4.861 *	8.448 **	+3.7-fold vs. +7-fold + 0 +3.7-fold vs. +7-fold *Antagonistic action*
**17**	-	-	-	-	-	-	-	-
**24**	13.15 **	NS	6.586 *	0 vs. −88 ^3^ + 0 0 vs. −88 *Antagonistic action*	21.11 ***	NS	NS	No interaction
**Collagen III/Collagen I Expression at the Protein Level**								
**3**	18.96 ***	NS	41.80 ***	+31 vs. +81 + (+47) +31 vs. +128 *Antagonistic action*	22.27 ***	NS	12.72 **	+58 vs. +77 + (+47) +58 vs. +124 *Antagonistic action*
**10**	94.62 ***	32.18 ***	92.83 ***	+1.32-fold vs. +2.5-fold + (+1.3-fold) +1.32-fold vs. +3.8-fold *Antagonistic action*	167.54 ***	13.65 **	32.73 ***	2.4-fold vs. +3.8-fold + (+1.3-fold) +2.4-fold vs. +5.1-fold *Antagonistic action*
**17**	5.054*	NS	6.845 *	0 vs. +21 + 0 0 vs. +21 *Antagonistic action*	20.59 ***	4.459 *	10.90 **	0 vs. +41 + 0 0 vs. +41 *Antagonistic action*
**24**	-	-	-	-	-	-	-	-

^1^ The findings of the two-way analysis of variance (ANOVA/MANOVA) are expressed as *F* values and the level of statistical significance (*p*). *F* values with *p* < 0.05 are considered statistically significant (* *p* < 0.05, ** *p* < 0.01, *** *p* < 0.001). NS—not statistically significant (*p* > 0.05). ^2^ In order to evaluate the possible character of the interaction between Cd and AE, the effect disclosed at their co-administration was compared to the sum of the effects noted at separate treatments with these agents (Cd + AE effect vs. Cd effect + AE effect). The Cd effect, AE effect, and Cd + AE effect are shown as percentage changes or factors of changes (+, increase; −, decrease) of a measured parameter vs. the control group. Cd–AE interaction was recognized as antagonistic when the result of simultaneous treatment with Cd and AE was less than the mathematic sum of the results noted at their separate applications. ^3^ The values represent percentage changes.

**Table 4:** The effect of *Aronia melanocarpa* L. berry extract (AE) on the concentrations of matrix metalloproteinase-1 (MMP-1) and matrix metalloproteinase-2 (MMP-2) in the serum of rats treated with cadmium (Cd) ^1,2^.

Group	Experiment Duration			
	3 Months	10 Months	17 Months	24 Months
**MMP-1 (ng/mL)**				
**Control**	12.94 ± 0.223	12.20 ± 0.196	14.58 ± 0.410	15.66 ± 0.563
**AE**	13.18 ± 0.481	12.14 ± 0.171	14.12 ± 0.349	16.39 ± 1.128
**Cd_1_**	12.21 ± 0.455	12.50 ± 0.141	16.04 ± 0.564 *	14.91 ± 0.626 **
**Cd_1_ + AE**	12.11 ± 0.737	11.57 ± 0.533	14.53 ± 0.404 ^†^	15.29 ± 0.841
**Cd_5_**	12.95 ± 0.229	10.85 ± 0.501	16.35 ± 0.351 **^,‡^	18.54 ± 0.370 **^,##^
**Cd_5_ + AE**	12.58 ± 0.197	12.09 ± 0.282	14.27 ± 0.504 ^††^	15.38 ± 0.355 ^††^
**MMP-2 (ng/mL)**				
**Control**	10.36 ± 0.452	8.368 ± 0.372	11.89 ± 0.486	11.25 ± 0.363
**AE**	12.81 ± 0.654	9.088 ± 0.579	15.36 ± 1.098	12.59 ± 0.764
**Cd_1_**	10.96 ± 0.663	10.07 ± 0.572	15.38 ± 0.590	15.77 ± 0.725 ^**^
**Cd_1_ + AE**	13.03 ± 0.513	9.698 ± 1.1391	14.17 ± 0.558	13.16 ± 0.679
**Cd_5_**	11.43 ± 0.646	10.48 ± 0.409	23.43 ± 1.841 ***^,##^	16.31 ± 0.561 ***
**Cd_5_ + AE**	11.15 ± 1.027	8.704 ± 0.645	12.56 ± 1.071 ^†††^	10.81 ± 0.929 ^††,#^

^1^ The animals were treated with Cd at the concentrations of 0, 1, and 5 mg Cd/kg in their diets and/or 0.1% aqueous AE. ^2^ Data are shown as mean ± SE for eight rats, except for seven animals in the AE, Cd_1_, and Cd_5_ groups after 24 months. Statistically significant differences (Kruskal–Wallis post hoc test): * p < 0.05, ** *p* < 0.01, and *** *p* < 0.001 vs. control group; ^†^ *p* < 0.05, ^††^ *p* < 0.01, and ^†††^ *p* < 0.001 vs. appropriate Cd group; ^‡^ *p* < 0.05 vs. AE group; ^#^ *p* < 0.05 and ^##^ *p* < 0.01 vs. appropriate group given the 1 mg Cd/kg diet (Cd_1_ group or Cd_1_ + AE group).

**Table 5:** The effect of *Aronia melanocarpa* L. berry extract (AE) on the concentrations of tissue metalloproteinase inhibitor-1 (TIMP-1) and tissue metalloproteinase inhibitor-2 (TIMP-2) in the serum of rats treated with cadmium (Cd) ^1,2^.

Group	Experiment Duration			
	3 Months	10 Months	17 Months	24 Months
**TIMP-1 (ng/mL)**				
**Control**	2.298 ± 0.059	2.216 ± 0.086	4.819 ± 0.170	2.624 ± 0.232
**AE**	2.158 ± 0.106	2.049 ± 0.169	3.968 ± 0.155	2.014 ± 0.209
**Cd_1_**	2.324 ± 0.156	1.971 ± 0.112	2.533 ± 0.135 ***^,‡^	1.469 ± 0.181 *
**Cd_1_ + AE**	2.061 ± 0.176	2.075 ± 0.075	3.780 ± 0.178 ^†^	2.152 ± 0.216 ^†^
**Cd_5_**	2.235 ± 0.113	2.136 ± 0.106	2.643 ± 0.104 ***^,‡^	1.342 ± 0.143 **
**Cd_5_ + AE**	2.460 ± 0.102	2.115 ± 0.094	3.229 ± 0.211 *^,†^	2.413 ± 0.226 ^†^
**TIMP-2 (ng/mL)**				
**Control**	1.722 ± 0.064	1.833 ± 0.027	1.622 ± 0.012	1.676 ± 0.048
**AE**	1.670 ± 0.010	1.712 ± 0.041	1.616 ± 0.027	1.730 ± 0.046
**Cd_1_**	1.548 ± 0.052	1.563 ± 0.085 *	1.589 ± 0.023	1.597 ± 0.068
**Cd_1_ + AE**	1.643 ± 0.013	1.855 ± 0.035 ^†^	1.720 ± 0.032 ^†^	1.728 ± 0.034
**Cd_5_**	1.317 ± 0.240	1.553 ± 0.019 **	1.613 ± 0.027	1.278 ± 0.064 *^,‡,##^
**Cd_5_ + AE**	1.652 ± 0.056	1.825 ± 0.065 ^†^	1.774 ± 0.041 ^†^	1.846 ± 0.048 ^†††,#^

^1^ The animals were treated with Cd at the concentrations of 0, 1, and 5 mg Cd/kg in their diets and/or 0.1% aqueous AE. ^2^ Data are shown as mean ± SE for eight rats, except for seven animals in the AE, Cd_1_, and Cd_5_ groups after 24 months. Statistically significant differences (Kruskal–Wallis post hoc test): * p < 0.05, ** *p* < 0.01, and *** *p* < 0.001 vs. control group; ^†^ *p* < 0.05 and ^†††^ *p* < 0.001 vs. appropriate Cd group; ^‡^ *p* < 0.05 vs. AE group; ^#^ *p* < 0.05 and ^##^ *p* < 0.01 vs. appropriate group given the 1 mg Cd/kg diet (Cd_1_ group or Cd_1_ + AE group).

**Table 6:** Mutual dependencies between the parameters of collagen metabolism in the liver ^1^.

PARAMETER	MMP-1 Concentration		MMP-2 Concentration	TIMP-1 Concentration	TIMP-2 Concentration
Total Collagen Concentration		0.233 ***	0.332 ***	NS	0.184 *
Collagen I mRNA Expression		0.163 *	0.313 ***	−0.280 ***	NS
Collagen III mRNA Expression		NS	0.498 ***	−0.460 ***	NS
Collagen I Protein Expression		NS	−0.285 ***	NS	NS
Collagen III Protein Expression		NS	−0.186 *	0.513 ***	0.325 ***
MMP-1 Concentration		-	0.448 ***	0.268 ***	0.603 ***
MMP-2 Concentration		-	-	NS	0.446 ***
TIMP-1 Concentration		-	-	-	0.498 ***

^1^ Data are expressed as *r* values and the level of statistical significance (*p*). The values of *r* with *p* < 0.05 were considered statistically significant (* *p* < 0.05, *** *p* < 0.001). NS—not statistically significant (*p* > 0.05). MMP-1, matrix metalloproteinase-1; MMP-2, matrix metalloproteinase-2; TIMP-1, tissue inhibitor of metalloproteinase-1; TIMP-2, tissue inhibitor of metalloproteinase-2.

## Supplementary Materials

The following are available online at https://www.mdpi.com/2072-6643/12/9/2766/s1, Table S1: The impact of exposure to cadmium (Cd) for 3–24 months and of *Aronia melanocarpa* L. berry extract (AE) co-administration on the morphological structure of the livers of rats; Table S2: Evaluation of the main and interactive effects of cadmium (Cd) and *Aronia melanocarpa* L. berry extract (AE) on the concentration of total collagen in the livers of rats; Table S3: Evaluation of the main and interactive effects of cadmium (Cd) and *Aronia melanocarpa* L. berry extract (AE) on the concentrations of matrix metalloproteinase-1 (MMP-1) and matrix metalloproteinase-2 (MMP-2) in the livers of rats; Table S4: Evaluation of the main and interactive effects of cadmium (Cd) and *Aronia melanocarpa* L. berry extract (AE) on the concentrations of tissue metalloproteinase inhibitor-1 (TIMP-1) and tissue metalloproteinase inhibitor-2 (TIMP-2) in the livers of rats; Table S5: Evaluation of the main and interactive effects of cadmium (Cd) and *Aronia melanocarpa* L. berry extract (AE) on the concentrations of matrix metalloproteinase-1 (MMP-1) and matrix metalloproteinase-2 (MMP-2) in the serum of rats; Table S6: Evaluation of the main and interactive effects of cadmium (Cd) and *Aronia melanocarpa* L. berry extract (AE) on the concentrations of tissue metalloproteinase inhibitor-1 (TIMP-1) and tissue metalloproteinase inhibitor-2 (TIMP-2) in the serum of rats; Table S7: The impact of *Aronia melanocarpa* L. berry extract (AE) on the content (µg) and the concentration (µg/g) of cadmium (Cd) in the livers of rats.

## Abbreviations

AE	extract from *Aronia melanocarpa* L. berries
bp	base pair
b.w.	body weight
ANOVA	one-way analysis of variance
ANOVA/MANOVA	two-way analysis of variance
Cd	cadmium
Cd^2+^	cadmium ion
cDNA	complementary deoxyribonucleic acid
CCl_4_	carbon tetrachloride
CV	coefficient of variation
ECM	extracellular matrix
FR	free radicals
HSC	hepatic stellate cells
MMP-1	matrix metalloproteinase-1
MMP-2	matrix metalloproteinase-2
MMPs	matrix metalloproteinases
mRNA	messenger ribonucleic acid
NaCl	sodium chloride
*p*	level of statistical significance
*r*	correlation coefficient
RNA	ribonucleic acid
ROS	reactive oxygen species
Real-Time PCR	real-time polymerase chain reaction
RT-PCR	reverse transcriptase polymerase chain reaction
SE	standard error
TGF-β1	trans-forming growth factor-β1
TIMPs	tissue inhibitors of metalloproteinases
TIMP-1	tissue inhibitor of metalloproteinase-1
TIMP-2	tissue inhibitor of metalloproteinase-2
TNF-α	tumor necrosis factor-α
*w/v*	weight/volume
vs.	versus
*v/v*	volume/volume
α-SMA	α-smooth muscle actin
