# Supplementary material for: The Impact of a Polyphenol-Rich Extract from the Berries of Aronia melanocarpa L. on Collagen Metabolism in the Liver: A Study in an In Vivo Model of Human Environmental Exposure to Cadmium
Source: Nutrients. 2020 Sep 10;12(9):2766. doi: 10.3390/nu12092766 (PMC7551016; doi:10.3390/nu12092766)
Supplement: Supplementary file 1 [file nutrients-12-02766-s001.pdf]

Supplementary Materials

Table S1. The impact of exposure to cadmium (Cd) for 3-24 months and *Aronia melanocarpa* L. berry extract (AE) co-administration on the morphological structure of the livers of rats. [1, 2.]

Group	Blurred Trabecular Structure of the Lobes	Microvascular Steatosis	Colliquative Necrosis	Vacuolization, Enlarged Dimensions of Cells	Mononuclear Cell Infiltrations
**3 Months**					
**Effect of exposure to the 1 mg Cd/kg diet**	sporadic change [3], in some cells in lobes	lack of change	lack of change	sporadic change, in almost all cells in lobes	sporadic change, in almost all cells in lobes
**Effect of AE co-administration**	no effect	no effect	no effect	partial protection (sporadic change, in some cells in lobes)	no effect
**Effect of exposure to the 5 mg Cd/kg diet**	sporadic change, in almost all cells in lobes	sporadic change, in almost all cells in lobes	sporadic change, in some cells in lobes	sporadic change, in almost all cells in lobes	sporadic change, in some cells in lobes
**Effect of AE co-administration**	partial protection (sporadic change, in some cells in lobes)	partial protection (sporadic change, in some cells in lobes)	complete protection	partial protection (sporadic change, in some cells in lobes)	no effect
**10 Months**					
**Effect of exposure to the 1 mg Cd/kg diet**	sporadic change, in almost all cells in lobes	sporadic change, in some cells in lobes	sporadic change, in single cells	sporadic change, in some cells in lobes	sporadic change, in some cells in lobes
**Effect of AE co-administration**	partial protection (sporadic change, in some cells in lobes)	complete protection	complete protection	partial protection (sporadic change, in single cells)	no effect
**Effect of exposure to the 5 mg Cd/kg diet**	in almost all cells in some lobes in a majority of animals [4]	sporadic change, in almost all cells in lobes	sporadic change, in almost all cells in lobes	sporadic change, in almost all cells in lobes	sporadic change, in almost all cells in lobes
**Effect of AE co-administration**	partial protection sporadic change, in almost all cells in lobes	partial protection (sporadic change, in some cells in lobes)	partial protection (sporadic change, in some cells in lobes)	partial protection (sporadic change, in some cells in lobes)	partial protection (sporadic change, in some cells in lobes)
**17 Months**					
**Effect of exposure to the 1 mg Cd/kg diet**	in almost all cells in some lobes in a majority of animals	in almost all cells in some lobes in a majority of animals	in almost all cells in some lobes in a majority of animals	in almost all cells in some lobes in a majority of animals	in almost all cells in some lobes in a majority of animals
**Effect of AE co-administration**	partial protection (sporadic change, in almost all cells in lobes)	partial protection (sporadic change, in almost all cells in lobes)	partial protection (sporadic change, in almost all cells in lobes)	partial protection (in some cells in some lobes in a majority of animals)	partial protection (sporadic change, in almost all cells in lobes)
**Effect of exposure to the 5 mg Cd/kg diet**	in almost all cells in some lobes in all animals	in almost all cells in some lobes in a majority of animals	in almost all cells in some lobes in all animals	in almost all cells in some lobes in all animals	in almost all cells in some lobes in a majority of animals
**Effect of AE co-administration**	partial protection (in almost all cells in some lobes in a majority of animals)	partial protection (sporadic change, in almost all cells in lobes)	partial protection (in almost all cells in some lobes in a majority of animals)	partial protection (in the majority of cells in some lobes in a majority of animals)	partial protection (sporadic change, in almost all cells in lobes)
**24 Months**					
**Effect of exposure to the 1 mg Cd/kg diet**	in almost all cells in some lobes in a majority of animals	in almost all cells in some lobes in all animals	in almost all cells in some lobes in a majority of animals	in almost all cells in some lobes in all animals	in almost all cells in some lobes in a majority of animals
**Effect of AE**	partial protection	partial protection	partial protection	partial protection	partial protection

**co-administration**	(sporadic change, in almost all cells in lobes)	(in almost all cells in some lobes in a majority of animals)	(sporadic change, in almost all cells in lobes)	(in some cells in some lobes in a majority of animals)	(sporadic change, in almost all cells in lobes)
**Effect of exposure to the 5 mg Cd/kg diet**	in almost all cells in some lobes in all animals	in almost all cells in some lobes in all animals	in almost all cells in some lobes in all animals	in some cells in some lobes in all animals	in almost all cells in some lobes in a majority of animals
**Effect of AE co-administration**	partial protection (in almost all cells in some lobes in a majority of animals)	partial protection (in almost all cells in some lobes in a majority of animals)	partial protection (in almost all cells in some lobes in a majority of animals)	partial protection (in some cells in some lobes in a majority of animals)	partial protection (sporadic change, in almost all cells in lobes)

[1] The animals were treated with Cd at the concentration of 1 and 5 mg Cd/kg diet alone or were co-administered with 0.1% aqueous AE. [2] The histological structure of the liver in the animals treated with Cd alone and co-administered with AE has been reported in detail elsewhere [12]. [3] The change occurred in one or two animals of a group. [4] The change occurred in 5–7 animals of a group.

Table S2. Evaluation of the main and interactive effects of cadmium (Cd) and *Aronia melanocarpa* L. berry extract (AE) on the concentration of total collagen in the livers of rats. [1.]

Duration (Months)	1 mg Cd/kg Diet + AE				5 mg Cd/kg Diet + AE			
	Main Effect of Cd	Main Effect of AE	Interactive Effect of Cd + AE	Cd + AE Effect vs. Cd Effect + AE Effect *Possible Character of Cd – AE Interaction*	Main Effect of Cd	Main Effect of AE	Interactive Effect of Cd + AE	Cd + AE Effect vs. Cd Effect + AE Effect *Possible Character of Cd – AE Interaction*
**Total collagen**								
**3**	NS	7.507*	NS	No interaction	19.29***	9.716**	NS	No interaction
**10**	7.277*	NS	NS	No interaction	-	-	-	-
**17**	-	-	-	-	-	-	-	-
**24**	-	-	-	-	NS	5.885*	NS	No interaction

[1] The results of the two-way analysis of variance (ANOVA/MANOVA) are expressed as *F* values and the level of statistical significance (*p*). *F* values having $p < 0.05$ were considered statistically significant (* $p < 0.05$, ** $p < 0.01$, *** $p < 0.001$). NS – not statistically significant ($p > 0.05$).

Table S3. Evaluation of the main and interactive effects of cadmium (Cd) and *Aronia melanocarpa* L. berry extract (AE) on the concentrations of matrix metalloproteinase-1 (MMP-1) and matrix metalloproteinase-2 (MMP-2) in the livers of rats. [1, 2.]

Duration (Months)	1 mg Cd/kg Diet + AE				5 mg Cd/kg Diet + AE			
	Main Effect of Cd	Main Effect of AE	Interactive Effect of Cd + AE	Cd + AE Effect vs. Cd Effect + AE Effect *Possible Character of Cd – AE Interaction*	Main Effect of Cd	Main Effect of AE	Interactive Effect of Cd + AE	Cd + AE Effect vs. Cd Effect + AE Effect *Possible Character of Cd – AE Interaction*
**MMP-1**								
**3**	NS	9.733**	NS	No interaction	-	-	-	-
**10**	NS	18.31***	15.90***	-26 [3] vs. +16 + 0<br>-26 vs. +16 [4]	-	-	-	-
**17**	NS	4.966*	6.198*	0 vs. +42 + 0<br>0 vs. +42<br>*Antagonistic action*	-	-	-	-
**24**	-	-	-	-	NS	7.062*	NS	No interaction
**MMP-2**								
**3**	NS	9.124**	NS	No interaction	-	-	-	-

**10**	NS	5.838*	NS	No interaction	22.66***	NS	NS	No interaction
**17**	NS	21.48***	NS	No interaction	NS	18.52***	NS	No interaction
**24**	5.102*	NS	NS	No interaction	NS	NS	NS	No interaction

[1] The results of the two-way analysis of variance (ANOVA/MANOVA) are expressed as *F* values and the level of statistical significance (p). *F* values having $p < 0.05$ were considered statistically significant (* $p < 0.05$, ** $p < 0.01$, *** $p < 0.001$). NS – not statistically significant ($p > 0.05$). [2] In order to evaluate the possible character of the interaction between Cd and AE, the effect disclosed at their co-administration was compared to the sum of the effects noted at separate treatment with these agents (Cd + AE effect vs. Cd effect + AE effect). Cd effect, AE effect, and Cd + AE effect are shown as percentage changes (+, increase; −, decrease) of a measured parameter vs. the control group. Cd − AE interaction was recognized as antagonistic when the result of simultaneous treatment with Cd and AE was lesser than the mathematic sum of the results noted at their separate application. [3] The values represent percentage changes. [4] The evaluation of the character of Cd – AE interaction was impossible.

Table S4. Evaluation of the main and interactive effects of cadmium (Cd) and *Aronia melanocarpa* L. berry extract (AE) on the concentrations of tissue metalloproteinase inhibitor-1 (TIMP-1) and tissue metalloproteinase inhibitor-2 (TIMP-2) in the livers of rats. [1.]

**Duration (Months)**	**1 mg Cd/kg Diet + AE**				**5 mg Cd/kg Diet + AE**			
	**Main Effect of Cd**	**Main Effect of AE**	**Interactive Effect of Cd + AE**	**Cd + AE Effect vs. Cd Effect + AE Effect** ***Possible Character of Cd – AE Interaction***	**Main Effect of Cd**	**Main Effect of AE**	**Interactive Effect of Cd + AE**	**Cd + AE Effect vs. Cd Effect + AE Effect** ***Possible Character of Cd – AE Interaction***
				**TIMP-1**				
**3**	-	-	-	-	-	-	-	-
**10**	-	-	-	-	NS	NS	NS	No interaction
**17**	NS	16.98***	NS	No interaction	-	-	-	-
**24**	-	-	-	-	-	-	-	-
				**TIMP-2**				
**3**	NS	NS	NS	No interaction	-	-	-	-
**10**	NS	6.593*	NS	No interaction	-	-	-	-
**17**	NS	11.78**	NS	No interaction	NS	16.53***	NS	No interaction
**24**	-	-	-	-	-	-	-	-

[1] The results of the two-way analysis of variance (ANOVA/MANOVA) are expressed as *F* values and the level of statistical significance (p). *F* values having $p < 0.05$ were considered statistically significant (* $p < 0.05$, ** $p < 0.01$, *** $p < 0.001$). NS – not statistically significant ($p > 0.05$).

Table S5. Evaluation of the main and interactive effects of cadmium (Cd) and *Aronia melanocarpa* L. berry extract (AE) on the concentrations of matrix metalloproteinase 1 (MMP-1) and matrix metalloproteinase 2 (MMP-2) in the serum of rats. [1,2]

	1 mg Cd/kg Diet + AE				5 mg Cd/kg Diet + AE			
**Duration (Months)**	**Main Effect of Cd**	**Main Effect of AE**	**Interactive Effect of Cd + AE**	**Cd + AE Effect vs. Cd Effect + AE Effect** ***Possible Character of Cd – AE Interaction***	**Main Effect of Cd**	**Main Effect of AE**	**Interactive Effect of Cd + AE**	**Cd + AE Effect vs. Cd Effect + AE Effect** ***Possible Character of Cd – AE Interaction***
				**MMP-1**				
**3**	-	-	-	-	-	-	-	-
**10**	-	-	-	-	-	-	-	-
**17**	4.607 *	4.981*	NS	No interaction	5.608 *	9.626**	NS	No interaction
**24**	NS	NS	NS	No interaction	NS	NS	8.688**	0 vs. +18 [3] + 0<br>0 vs. 18<br>*Antagonistic action*
				**MMP-2**				
**3**	-	-	-	-	-	-	-	-
**10**	-	-	-	-	-	-	-	-
**17**	-	-	-	-	15.77***	NS	9.458**	0 vs. +97 + 0<br>0 vs. 97<br>*Antagonistic action*
**24**	12.76**	9.159**	34.42***	0 vs. +40 + 0<br>0 vs. 40<br>*Antagonistic action*	5.608*	8.970**	24.25***	0 vs. +45 + 0<br>0 vs. 45<br>*Antagonistic action*

[1] The results of the two-way analysis of variance (ANOVA/MANOVA) are expressed as *F* values and the level of statistical significance (p). *F* values having $p < 0.05$ were considered statistically significant (* $p < 0.05$, ** $p < 0.01$, *** $p < 0.001$). NS – not statistically significant ($p > 0.05$). [2] In order to evaluate the possible character of the interaction between Cd and AE, the effect disclosed at their co-administration was compared to the sum of the effects noted at separate treatment with these agents (Cd + AE effect vs. Cd effect + AE effect). Cd effect, AE effect, and Cd + AE effect are shown as percentage changes (+, increase) of a measured parameter vs. the control group. Cd − AE interaction was recognized as antagonistic when the result of simultaneous treatment with Cd and AE was lesser than the mathematic sum of the results noted at their separate application. [3] The values represent percentage changes.

Table S6. Evaluation of the main and interactive effects of cadmium (Cd) and *Aronia melanocarpa* L. berry extract (AE) on the concentrations of tissue metalloproteinase inhibitor-1 (TIMP-1) and tissue metalloproteinase inhibitor-2 (TIMP-2) in the serum of rats. [1,2]

**Duration (Months)**	**1 mg Cd/kg Diet + AE**				**5 mg Cd/kg Diet + AE**			
	**Main Effect of Cd**	**Main Effect of AE**	**Interactive Effect of Cd + AE**	**Cd + AE Effect vs. Cd Effect + AE Effect** ***Possible Character of Cd – AE Interaction***	**Main Effect of Cd**	**Main Effect of AE**	**Interactive Effect of Cd + AE**	**Cd + AE Effect vs. Cd Effect + AE Effect** ***Possible Character of Cd – AE Interaction***
				**TIMP-1**				
**3**	-	-	-	-	-	-	-	-
**10**	-	-	-	-	-	-	-	-
**17**	59.54***	NS	42.86***	0 vs. -44 [3] + 0<br>0 vs. -44<br>*Antagonistic action*	78.49***	NS	19.09***	0 vs. -45 + (-33)<br>0 vs. -78<br>*Antagonistic action*
**24**	5.375*	NS	8.785**	0 vs. -43 + 0<br>0 vs. -43<br>*Antagonistic action*	4.245*	NS	16.35***	0 vs. -49 + 0<br>0 vs. -49<br>*Antagonistic action*
				**TIMP-2**				
**3**	-	-	-	-	-	-	-	-
**10**	NS	NS	15.62***	0 vs. -15 + 0<br>0 vs. -15<br>*Antagonistic action*	NS	NS	22.10***	0 vs. -10 + 0<br>0 vs. -10<br>*Antagonistic action*
**17**	-	-	-	-	-	-	-	-
**24**	-	-	-	-	7.566*	36.55***	24.97***	0 vs. -24 + 0<br>0 vs. -24<br>*Antagonistic action*

[1] The results of the two-way analysis of variance (ANOVA/MANOVA) are expressed as *F* values and the level of statistical significance (*p*). *F* values having $p < 0.05$ were considered statistically significant (* $p < 0.05$, ** $p < 0.01$, *** $p < 0.001$). NS – not statistically significant ($p > 0.05$). [2] In order to evaluate the possible character of the interaction between Cd and AE, the effect disclosed at their co-administration was compared to the sum of the effects noted at separate treatment with these agents (Cd + AE effect vs. Cd effect + AE effect). Cd effect, AE effect, and Cd + AE effect are shown as percentage changes (−, decrease) of a measured parameter vs. the control group. Cd − AE interaction was recognized as antagonistic when the result of simultaneous treatment with Cd and AE was lesser than the mathematic sum of the results noted at their separate application. [3] The values represent percentage changes.

Table S7. The impact of *Aronia melanocarpa* L. berry extract (AE) on the content (μg) and the concentration (μg/g) of cadmium (Cd) in the livers of rats. [1, 2, 3.]

Duration (Months)	1 mg Cd/kg Diet			5 mg Cd/kg Diet		
	Cd group	Effect of Cd + AE	Effect of AE	Cd group	Effect of Cd + AE	Effect of AE
			Content of Cd			
**3**	1.0920 ± 0.207***	↑ 2.8-fold	↙ 29%	7.510 ± 1.471***	↑ 20-fold	↙ 25%
**10**	1.819 ± 0.754***	↑ 8.4-fold	⇔	17.061 ± 1.812***	↑ 64-fold	↙ 24%
**17**	2.147 ± 0.235***	↑ 13-fold	⇔	24.066 ± 6.072***	↑ 144-fold	↙ 28%
**24**	4.442 ± 0.912***	↑ 17-fold	↙ 34%	39.922 ± 3.725***	↑ 145-fold	↙ 36%
			Concentration of Cd			
**3**	0.145 ± 0.009***	↑ 2.8-fold	↙ 33%	0.912 ± 0.053***	↑ 21-fold	↙ 18%
**10**	0.199 ± 0.028***	↑ 7.8-fold	⇔	1.617 ± 0.112***	↑ 62-fold	↙ 11%
**17**	0.211 ± 0.019***	↑ 14-fold	⇔	2.449 ± 0.178***	↑ 134-fold	↙ 24%
**24**	0.364 ± 0.025***	↑ 17-fold	↙ 37%	2.755 ± 0.089***	↑ 182-fold	↙ 10%

[1] The animals were treated with Cd at the concentration of 0, 1, and 5 mg Cd/kg diet and/or 0.1% aqueous AE. [2] Table presents Cd content and concentration and changes in its concentration ($p < 0.05$; ANOVA, Duncan's multiple range post hoc test) compared to the control group (*** $p < 0.001$): a percentage or factor of increase (↑) and the appropriate Cd group: a percentage increase (↗), decrease (↙), or lack of statistically significant change (⇔; $p > 0.05$) are indicated. Cd content and concentration in control groups (mean ± SE) ranged from 0.152 ± 0.601 to 0.272 ± 0.083 μg and 0.0137 ± 0.0015 to 0.0348 ± 0.0026 μg/g, respectively. [3] Detailed data on the impact of AE on the content and the concentration of Cd in the liver of rats exposed to Cd have already been published [17].
